# Multi‐Channel Electrically Tunable Varifocal Metalens With Compact Multilayer Polarization‐Dependent Metasurfaces and Liquid Crystals

**DOI:** 10.1002/advs.75483

**Published:** 2026-05-04

**Authors:** Zhiyao Ma, Yize Liu, Zhe Li, Tian Tian, Yuxuan Liao, Xue Feng, Yongzhuo Li, Kaiyu Cui, Fang Liu, Hao Sun, Wei Zhang, Yidong Huang

**Affiliations:** ^1^ Department of Electronic Engineering Tsinghua University Beijing China

## Abstract

As an essential module of optical systems, a varifocal lens usually consists of multiple mechanically moving lenses along the optical axis. The recent development of metasurfaces with tunable functionalities holds the promise of miniaturizing varifocal lenses. However, existing varifocal metalenses are hard to combine electrical tunability with a scalable number and range of focal lengths, which limits the practical applications. Our previous work shows that the electrically tunable channels could be increased to 2*^N^* by cascading *N* polarization‐dependent metasurfaces with liquid crystals (LCs). Here, we demonstrate a compact eight‐channel electrically tunable varifocal metalens with three single‐layer polarization‐multiplexed bi‐focal metalens and three LC cells. The total thickness of the device is ∼6 mm, while the focal lengths can be switched among eight values within the range of 3.6–9.6 mm (3.6, 4.2, 4.5, 4.9, 6.2, 6.9, 8.4, 9.6 mm). The scheme is scalable in both the number and range of focal lengths, as well as has the advantage of further miniaturization. We believe that our proposal would open new possibilities for miniaturized imaging systems, AR/VR displays, LiDAR, etc.

## Graphical abstract

A compact eight‐channel electrically tunable varifocal metalens with three single‐layer polarization‐multiplexed bi‐focal metalens and three LC cells. The total thickness of the device is ∼6 mm, while the focal lengths can be switched among eight values (3.6, 4.2, 4.5, 4.9, 6.2, 6.9, 8.4, and 9.6 mm).

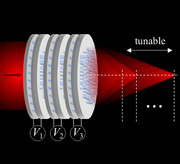

## 1 Introduction

Varifocal lens is an essential component in optical imaging and display systems. Usually, the required phase gradient for a lens is achieved by lightwave propagation through a curved, bulky transparent material, while multiple mechanically moving lenses along the optical axis are required to vary the focal length. Consequently, it is a great challenge to miniaturize the varifocal lens for practical applications.

Metasurface is developed by arranging subwavelength scatters in a plane for controlling the property of lightwave, including phase [1, 2], amplitude [3, 4, 5], polarization [6, 7], frequency [8, 9], etc. Metasurface lens (also called metalens) [10, 11, 12, 13, 14] can achieve the phase gradient of a lens within subwavelength thickness and resolution, thus possessing higher compactness, higher numerical aperture (N.A.) [15, 16], and even richer functionalities [17, 18, 19] compared with the traditional lens. Except for mechanically moving multiple layers along the optical axis [20], the focal length of metalenses could be tuned by various mechanisms, including reconfigurable metasurface [21, 22, 23, 24, 25], mechanically stretching [26, 27, 28], cascaded Moiré metalens [29, 30, 31], orbital angular momentum (OAM) multiplexing [32, 33], etc. However, pixel‐by‐pixel active reconfigurable metalenses mainly operate in the microwave regime [25], since reconfiguring optical subwavelength structures would face significant technical difficulty. Mechanically stretched metalens [26, 27, 28] and Moiré metalens [29, 30, 31] still rely on mechanically moving, which limits the speed and stability of the device. Other reconfiguring mechanisms, such as phase change materials [21, 22, 23] and thermo‐optical effects [24] are usually limited in number or range of focal lengths.

Besides these methods, liquid crystals (LCs) offer a promising approach for stable, high‐speed, and electrically tunable varifocal metalenses at optical frequency. One scheme is to encapsulate LC on the metasurface, so that the local phase response of meta‐atoms can be modified through electrically controlling the refractive index of LC [34, 35]. Thus, the focal length can be continuously tuned. Another scheme is to exploit the polarization degree of freedom (DoF) of light, thereby switching between two discrete focal lengths [36, 37, 38, 39, 40]. However, the range of continuously tunable focal length is limited to ∼20% due to the refractive index range of LC, while the discrete switchable focal lengths are limited to two due to the number of polarization states. To combine a scalable range and number of focal lengths, a possible solution is cascading multiple polarization‐dependent metasurfaces for more DoFs. Specifically, we have recently proposed an *N*‐layer cascaded structure of polarization‐dependent metasurfaces and LCs for 2*^N^* electrically switchable channels of vortex beam generation and beam steering [41].

In this work, our previous proposal is extended to a compact 2*^N^*‐channel electrically tunable varifocal metalens with *N* single‐layer polarization‐multiplexed bi‐focal metalens and *N* LC cells. By controlling the input polarization states through an LC cell, the focal length of each single‐layer metalens can be switched between two values. Then, the equivalent focal length of an *N*‐layer cascaded metalens can be switched in 2*^N^* values, corresponding to the 2*^N^* combinations of input polarization states at each single‐layer metalens. The number, resolution, and range of focal lengths would be scalable with an increased number of layers. Experimentally, by alternately stacking three single‐layer metalenses and three LC cells, we have demonstrated a varifocal metalens with a thickness of ∼6 mm and eight electrically switchable channels. The focal lengths can be switched among eight values within the range of 3.6 to 9.6 mm (3.6, 4.2, 4.5, 4.9, 6.2, 6.9, 8.4, 9.6 mm). The focused FWHM (full‐width at half‐maximum) is within the range of 19.1 to 40.6 µm, while the efficiency is within the range of 5.4% to 10.3%.

## 2 Results

**FIGURE 1:**
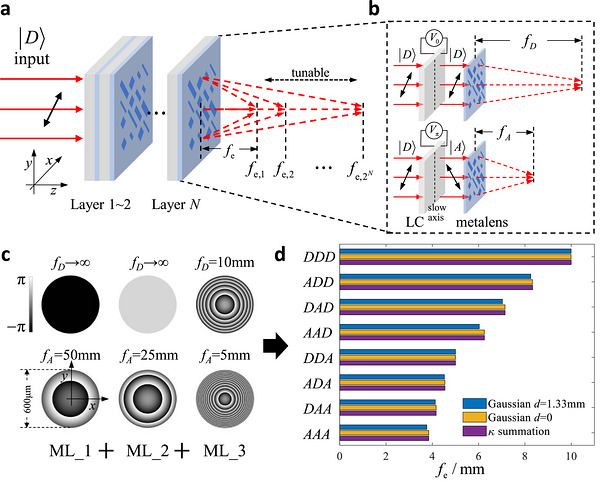
Principle and design of compact metalens. (a) Schematic of compact metalens with alternately cascaded *N* single‐layer metalenses (ML) and *N* LC cells. (b) One layer of the cascaded structure. An LC cell is attached on the input side of a polarization‐multiplexed bifocal metalens, so that the focal length can be switched between *f_D_* and *f_A_* according to the input states |*D*〉 and |*A*〉. (c) Phase profiles for |*D*〉 and |*A*〉 input of each single‐layer metalens with the corresponding focal lengths. (d) Calculated whole focal lengths corresponding to eight input polarization combinations. Results by Gaussian beam propagation with *L*_exp_ = 1.33 mm, *L* = 0, and phase profile summation are compared.

Figure 1a is the schematic of a compact metalens with alternately cascaded *N* layers, while each layer consists of a metasurface and an LC cell attached on the input side, as shown in Figure 1b. The metasurface is designed as a polarization‐multiplexed bifocal metalens, where two lens phase profiles with different focal lengths are modulated on the input lightwave with two orthogonal polarization states, respectively [36, 37]. Here, the orthogonal input states at each bifocal metalens are designed as diagonal and anti‐diagonal linear polarization (denoted as |*D*〉 and |*A*〉), which can be manipulated by the vertical slow axis of LC. Specifically, the input state will be varied from one to the other with the π phase retardance, while it will remain constant with 0 phase retardance. Figure 1b shows the case of |*D*〉 input without loss of generality. Thus, the focal lengths of each single‐layer metalens can be independently switched between *f_D_* and *f_A_*.

After cascading *N* such layers, the total combinations of the input polarization state at each single‐layer metalens will be 2*^N^*. Supposing that all metalenses were attached with a sufficiently close distance, the functionality of the whole cascaded structure can be expressed as the summation of the phase profile loaded on each single‐layer metalens according to the input state. Here, the phase profile of each single lens is expressed in spatial coordinates (*x*,*y*) as Equation (1) [42], where *λ* is the wavelength and *f* is the focal length. Equation (1) is a linear expression of 1/*f*, thus the summation of multiple profiles is still a lens profile with a different focal length, as shown in Equation (2), which is a necessary condition for the following design of 2*^N^* channels [41].

(1) $$\varphi \left(x,y,f\right)=-\frac{\pi \left(x^{2}+y^{2}\right)}{\lambda f}$$

(2) $$\begin{array}{c} \varphi_{\mu }=\sum_{j=1}^{N}\varphi_{j,\mu_{j}}=\sum_{j=1}^{N}\left(-\frac{\pi \left(x^{2}+y^{2}\right)}{\lambda f_{j,\mu_{j}}}\right)=-\frac{\pi \left(x^{2}+y^{2}\right)}{\lambda }\sum_{j=1}^{N}\frac{1}{f_{j,\mu_{j}}} \end{array}$$

(3) $$f_{e,\mu }=1/\sum_{j=1}^{N}\frac{1}{f_{j,\mu_{j}}}$$

In Equation (2), **µ** is a *N*×1 vector representing the input polarization state at each single‐layer metalens. It can be seen that the functionality of the whole structure is still a lens, and the equivalent focal length of the whole cascaded structure *f*_e_ can be expressed in summation of reciprocal forms as Equation (3). Thus, the equivalent focal lengths can take 2*^N^* distinct values according to 2*^N^* values of **µ**. It should be noted that Equation (1) is chosen as the parabolic phase profile instead of the commonly used hyperbolic phase profile (see Equation S1 in Note S1) [10, 11, 12, 13] to meet the condition that the phase profile is a linear expression of certain parameters. Actually, the parabolic lens profile is almost equivalent to the commonly used hyperbolic phase profile for the current demonstration, since the N.A. is low and only normal incidence is considered. For more general cases, although a parabolic phase profile would introduce spherical aberration, it has been proven to exhibit a wider field‐of‐view (FOV) for imaging compared with a hyperbolic phase profile [43].

Moreover, the thickness of substrates and liquid crystals has to be considered for a practical implementation. Instead of simply summing the phase profiles of single‐layer metalenses, the theoretical modeling of the whole structure should include lightwave propagation between adjacent metalenses. Here, since paraxial approximation is satisfied in the demonstration, *q* parameters of the Gaussian beam are utilized to model the cascaded lens profiles with proper distance [44]. Details of the Gaussian beam propagation model can be found in Methods and Note S2.

With the Gaussian beam propagation model, the parameters of a three‐layer cascaded metalens can be properly designed. We have found that the focal length of the last‐layer metalens should be close to the expected cascaded focal lengths, while the focal lengths of former layers should be much larger. In this way, the propagation effect after former layers is not significant, so that the expected eight focal lengths can be distributed with a relatively smooth trend. Figure 1c shows the designed focal lengths for |*D*〉 and |*A*〉 input of each single‐layer metalens with the corresponding phase profiles. It should be mentioned that a lens with *f*→∞ corresponds to a uniform phase profile, which actually carries out no operation on the wavefront. Figure 1d shows the calculated eight focal lengths corresponding to the polarization combinations of each layer. For example, *DAA* represents that the input state at the first single‐layer metalens is |*D*〉, while the input state at the second and third single‐layer metalens are |*A*〉. The equivalent propagation distance between two adjacent single‐layer metalenses is set as *L*_exp_ = 1.33 mm according to the experiments (see Equation S13). Then, the calculated results by Gaussian beam propagation (Equations (4), (5), (6), (7)) with *L*_exp_ = 1.33 mm and *L* = 0, as well as phase profile summation (Equation 3), are compared. It can be seen that the propagation results with *L*_exp_ = 1.33 mm differ from summation results, while the propagation results with *L* = 0 are almost consistent with summation results. The Gaussian beam propagation model with *L* = 0 reduces to phase profile summation correctly.

**FIGURE 2:**
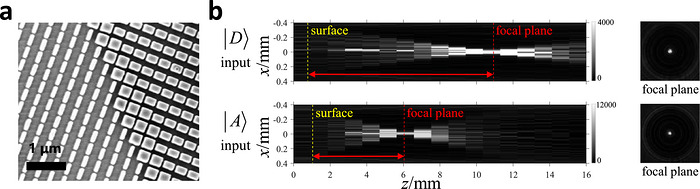
Characterization and measurement of single‐layer metalens. (a) SEM image of single‐layer metalens without spin‐coating. (b) Measured intensity profiles of the third metalens with |*D*〉and |*A*〉 input. Left column: *x*‐*z* intensity, right column: *x*‐*y* intensity at the focal plane.

To implement the cascaded metalens, first, the single‐layer metalenses are designed and fabricated by *α*‐Si nanopillars with a height of 500 nm at the operating wavelength of 780 nm. The SEM image of the fabricated metalens sample without spin‐coating is shown in Figure 2a. To protect the nanopillars, SU‐8 2002 photoresist with a thickness of ∼3 µm is spin‐coated as a spacer layer. Thus, the total thickness *d*_ML_∼0.54 mm of one single‐layer metalens mainly consists of the SiO_2_ substrate. Besides, the metalens diameter of 600 µm, so that paraxial approximation is satisfied since all of the focal lengths are more than 3 mm. Detailed design and fabrication process of single‐layer metalens are presented in the section of Methods, and more information can be found in Note S3.

The functionality of each single‐layer metalens is verified by measuring the focusing properties. In our scheme, the designed focal lengths of the former layers are much longer than the final tunable range, while the focal lengths of the third single‐layer metalens are within the final range. In other words, the focusing ability is mainly contributed by the third metalens. Therefore, we primarily characterized the third metalens. Figure 2b shows the replotted *x*‐*z* intensity profiles of the third metalens, along with the *x*‐*y* intensity profiles at focal planes. Since the intensity profile is zoomed by the measurement imaging system, the profiles in Figure 2b are rescaled to the real size. The measured focal lengths with |*D*〉 and |*A*〉 input are 10.2 and 5.0 mm, respectively, agreeing with the designed values of 10 and 5 mm. Furthermore, the imaging performance of the third metalens is measured for future comparison, which would be provided in the following sections. Details of the measurement process can be found in Methods, while the optical setup and results of the other single‐layer metalenses can be found in Note S4.

**FIGURE 3:**
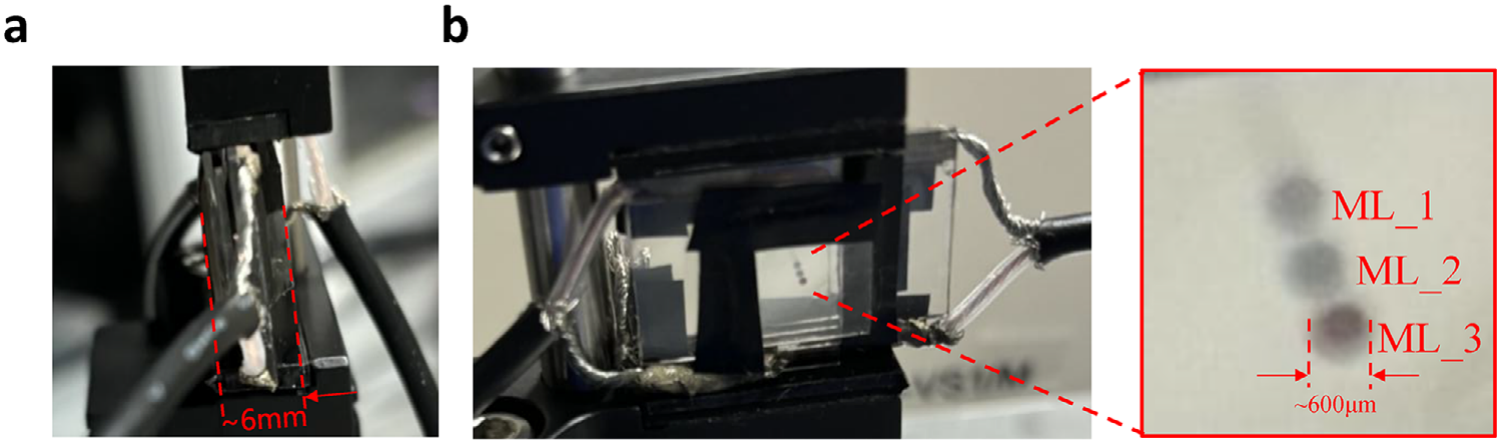
Implementation of three‐layer cascaded metalens. (a) Side view of the three‐layer cascaded metalens. (b) Normal view of the three‐layer cascaded metalens. Inset is a close view of three single‐layer metalenses.

Next, the LC cells filled with nematic LC molecules are customized from JCOPTIX. The total thickness of an LC cell is *d*_LC_∼1.45 mm, which mainly consists of the two glass substrates since the thickness of LC molecules is only 12 µm. Then, the cascaded metalens is implemented by alternately stacking three LC cells and three single‐layer metalenses with parameters shown in Figure 1b. The calibration of each LC cell and alignment of each single‐layer metalens are conducted during the corresponding step of stacking. The photograph of the implemented sample is shown in Figure 3a,b. As seen in the side view of Figure 3a, the total thickness of the cascaded metalens is ∼6 mm, which agrees with the summation of the thickness of all devices. The inset of Figure 3b clearly shows the effective regions of the three single‐layer metalenses with a diameter of ∼600 µm, whose centers are aligned along a line. The final alignment error in *x*‐*y* plane is less than 34 µm. Full parameters and the calibration process of the LC cell can be found in Note S5, while details of the stacking and alignment process can be found in Note S6.

**FIGURE 4:**
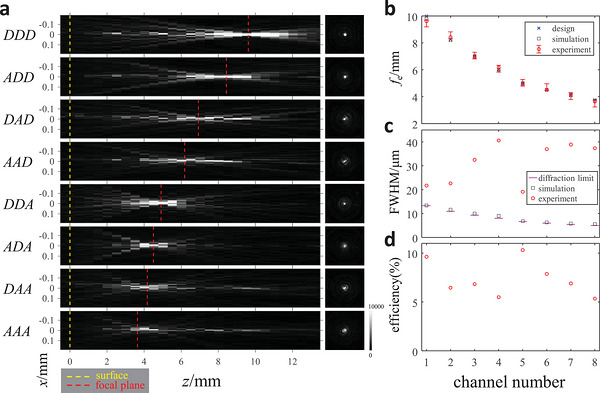
Cascaded metalens measurement. (a) Measured intensity profiles of eight channels. The left column is replotted *x*‐*z* intensity profiles, where the output plane and focal plane are marked by yellow and red dashed lines, respectively. The right column is original *x*‐*y* intensity profiles at the focal planes. Each profile is rescaled to the size at objective plane. (b) Comparison of measured, simulation, and designed focal lengths of eight channels. (c) Comparison of measured, simulation, and diffraction‐limited (0.51*λ*/NA) FWHM values of eight channels. (d) Measured efficiency of eight channels. In (b,c,d), the channel numbers 1 to 8 represent *DDD* to *AAA* as shown in (a), respectively.

The focusing property of cascaded metalens is measured with a similar optical setup for single metalens (see Figure S4 of Note S4.1). Here, the input beam is fixed on |*D*〉 since the polarization states are switched by LCs. For all eight channels, the replotted *x*‐*z* intensity profiles and original *x*‐*y* intensity profiles at the focal planes are shown in Figure 4a, respectively. It can be seen from Figure 4a that the measured intensity profiles and corresponding focal lengths of eight channels are 3.6, 4.2, 4.5, 4.9, 6.2, 6.9, 8.4, and 9.6 mm, respectively. Then, as shown in Figure 4b, the trend of the measured focal lengths agrees with the designed values, where the errors are less than 3.8%. The simulated focal lengths are also provided in Figure 4b. In addition, the FWHM and efficiency are measured and plotted in Figure 4c,d, respectively. The FWHM at the focal planes is obtained by 2D Gaussian fitting, which is within the range of 19.1 to 40.6 µm. For comparison, the simulated and diffraction‐limited (0.51*λ*/NA) FWHM values are also presented in Figure 4c. The simulated FWHM is at most 17% larger than the diffraction limit, while the measured FWHM is much larger. In addition, the FWHM of the third single metalens is measured as 27.2 and 33.8 µm for |*D*〉 and |*A*〉 input, which are also much larger than the diffraction limit. These indicate that the intrinsic aberration through cascading is moderate, while most aberration comes from single metalens, scattering at substrates, and other factors. The efficiency is defined as the ratio of the total intensity around the focal spot to the intensity of the input, which is within the range of 5.4% to 10.3%. As shown in Figure 4d, the efficiency of Channel 1 and 5 (corresponding to *DDD* and *DDA* polarization) is higher than that of other channels. This is mainly because the |*D*〉 channels of the first two layers are expected to be uniform phase profile, so that the transmittance *t* will be higher than that of |*A*〉 channel. Specifically, the measured (*t_D_*, *t_A_*) is (61.7%, 30.5%) for the first metalens and (85.1%, 52.4%) for the second metalens. Details of the measurement and data processing can be found in Note S7, while details of the simulation can be found in Note S8.3.

**FIGURE 5:**
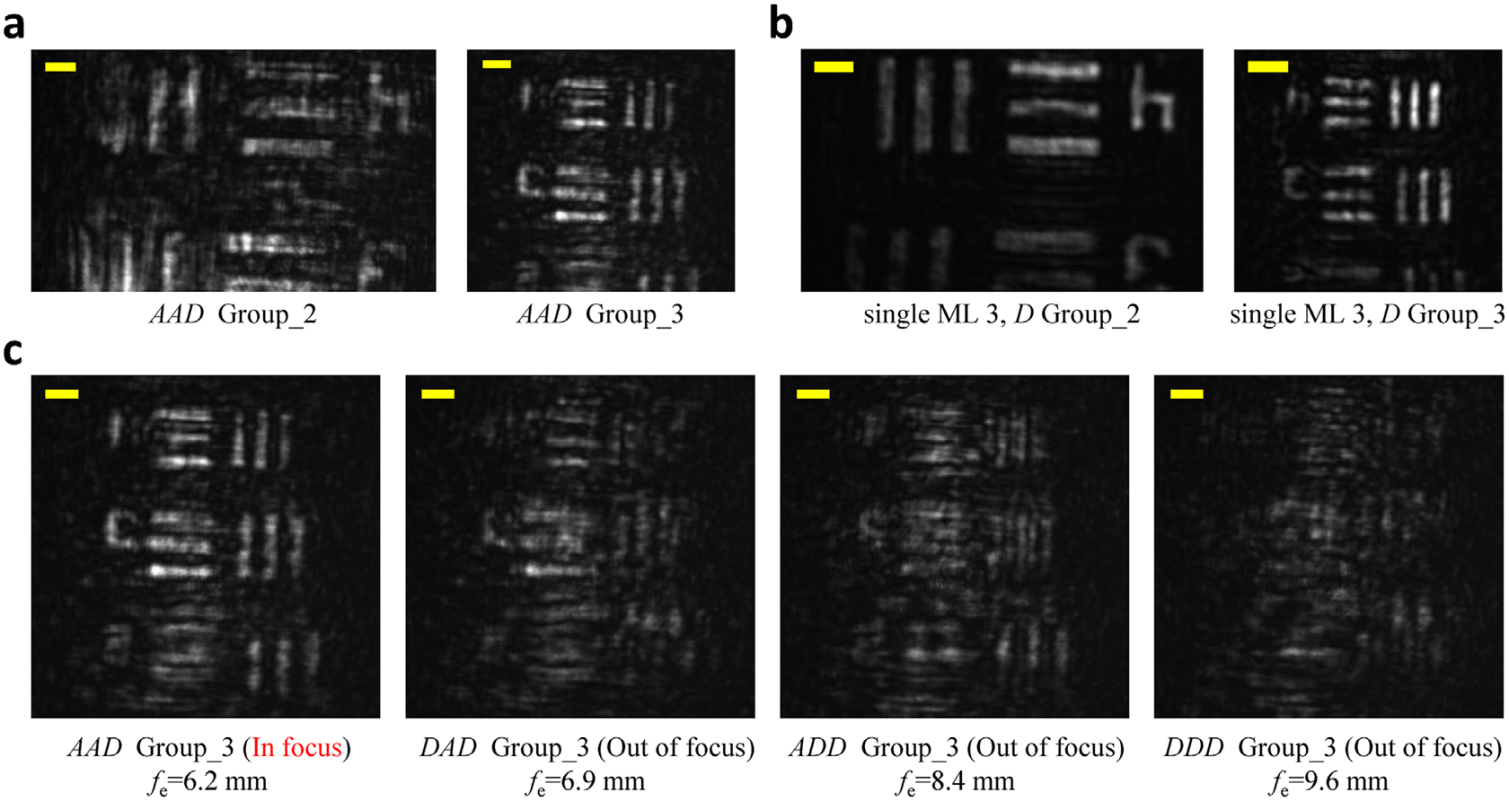
Cascaded metalens imaging demonstration. (a,b) Imaged picture comparison of cascaded and single metalens. (a) Group 2 and 3 under the *AAD* channel, *f*_e_ = 6.2 mm. (b) Imaged pictures of Group 2 and 3 under the |*D*〉 channel of the third single metalens, *f* = 10 mm. (c) In‐focus and out‐of‐focus image of Group 3 under switched channels of the cascaded metalens. All elements are fixed in location for in‐focus image of *f*_e_ = 6.2 mm. The corresponding effective focal lengths (*f*_e_) are noted. All scale bars are 100 µm at the imaging plane. The second panel in (a) is a rescaled version of the first panel in (c), displayed here for direct comparison.

Then, the imaging ability of the cascaded metalens is demonstrated with a negative USAF 1951 target. The imaged pictures of Group 2 and 3 under the *AAD* channel (*f*_e_ = 6.2 mm) are shown in Figure 5a. For comparison, the imaging results are recorded at the same regions of the third single metalens and presented in Figure 5b. It can be seen that the objects can still be resolved after cascading, while the noise is more significant since the efficiency is deteriorated. Then, the object, device, and image plane are fixed in location, and the channel is switched to other focal lengths. Imaged pictures under other channels are shown in Figure 5c. As expected, the object is no longer resolvable since the focal length changes. Additionally, the area of the imaged object is ∼1 mm in Figure 5c according to the real size of USAF 1951 elements, while the object distance is ∼15 mm according to the zoom ratio. It can be estimated that the current field of view (FOV) is at least 4°. The optical setup for imaging measurement can be found in Figure S6 of Note S4.2.

**FIGURE 6:**
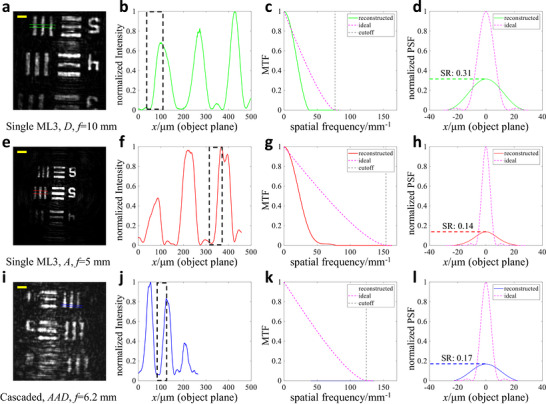
Imaging performance comparison of single and cascaded metalens. (a–d) Imaging performance of the third single metalens under |*D*〉 input. (a) Imaging of USAF 1951 Group 2. (b) Normalized intensity sampled along a line across Group 2, Element 5. The black dashed rectangle marks the region of interest for the Edge Spreading Function (ESF). (c) Reconstructed Modulation Transfer Function (MTF) from the ESF, and the diffraction‐limited ideal MTF. (d) Reconstructed Point Spreading Function (PSF) from MTF, and the diffraction‐limited ideal PSF. The Strehl ratio (SR) is denoted. (e–h) Corresponding results of third single metalens under |*A*〉 input. Sampled on Group 2, Element 5. (i–l) Corresponding results of the *AAD* channel of cascaded metalens. Sampled on Group 3, Element 4. All scale bars in (a,e,i) are 100 µm at the imaging plane.

Furthermore, the imaging performance is quantitatively studied. Take the |*D*〉 input image of third single metalens as an example. First, the intensity along a line across the USAF 1951 Group 2, Element 5 is sampled as shown in Figure 6a,b. According to the rising edge marked by the dashed rectangle in Figure 6b, the Edge Spreading Function (ESF) can be obtained. According to the ESF data, the Modulation Transfer Function (MTF) and the Point Spreading Function (PSF) can be reconstructed, which are shown in Figure 6c,d, respectively. The Strehl ratio can be obtained through the maximum value of PSF and the maximum value of diffraction‐limited ideal PSF. It can be seen that each reconstructed MTF cutoffs at around 40–50 mm^−1^, showing similar resolution. Comparing the cascaded *AAD* channel with the *D* input of the third single metalens, the Strehl ratio degraded from 0.31 to 0.17 through cascading. Details of the reconstruction process can be found in Note S4.2.

## 3 Discussion

According to the simulated and measured results of the three‐layer cascaded structure, the focal‐length range, FWHM, and imaging ability are mainly determined by the third single metalens. We have also conducted simulations to analyze the aberration from alignment error. The simulation results show that the alignment aberration will cause the FWHM to be only at most 5% larger than the diffraction limit. In addition, we have analyzed the composition of the efficiency and found that the total efficiency can be estimated as a product of the transmittance of each former layer of metalens or LC, and the focusing efficiency of the last metalens. Details can be found in Note S8.

Therefore, our demonstration shows the cascading of multiple polarization‐multiplexed bifocal metalenses can increase the number of electrically switchable focal lengths, while preserving most performance of single metalens, including range of focal length, FWHM, efficiency, and imaging ability. Although the current demonstration is a proof of principle and the performance is not particularly optimized, the performance of our cascading scheme can be optimized by using single metalens with improved performance. There is no inherent weakness in our scheme regarding these characteristics. More specifically, the first issue is that the design and fabrication process of single metalens can be improved for lower FWHM and higher efficiency. The approaches of P‐B phase [11, 12, 13, 14], inverse design [45, 46], etc., can be introduced. Second, the reflection at multiple substrates of LC cells and metasurfaces can be further reduced by anti‐reflection coating. Third, the alignment of cascaded structure can be performed under a mounting system [47] to reduce damage and achieve subwavelength precision.

Besides, to achieve more stacked layers and further miniaturization in practical applications, the thickness can be further reduced by improving the fabrication methods. The current thickness per layer is ∼2 mm, which is mainly composed of the substrates of LC cell and metasurface. According to a previous report, the total thickness of the LC cell could be reduced to about 50 µm [48]. Furthermore, LCs could be packaged on the substrate of metasurfaces for a fully integrated scheme [49]. Reduced thickness is also crucial to leverage the wide FOV of the parabolic profile (see Equation 1), since the alignment could be better preserved at obliquely incident.

Furthermore, other properties are also potentially improved by using single metalens with corresponding approaches, e.g., elimination of chromatic aberration for broadband response [11, 12, 13]. Thus, no additional layers would be required for achromatic response, while the efficiency and complexity can be mostly preserved by P‐B phase and dispersion engineering [11, 12, 13]. In addition, although in this work we utilized LC for switching the polarization states, LC is not essential. In fact, other tuning mechanisms for similar dual‐channel switchable metasurface layer could be utilized. For example, the platforms of thin‐film lithium niobate or phase‐change materials have the potential to achieve higher speed or efficiency than the current demonstration. Further description of the performance and potential enhancing approaches can be found in Note S8, along with a comprehensive list of reported varifocal metalens.

To conclude, a compact eight‐channel electrically tunable varifocal metalens is demonstrated by alternately stacking three single‐layer polarization‐multiplexed bi‐focal metalens and three LC cells. The total thickness is ∼6 mm, while the focal lengths are switchable among eight values within the range of 3.6–9.6 mm (3.6, 4.2, 4.5, 4.9, 6.2, 6.9, 8.4, 9.6 mm). Our scheme is scalable in resolution and range of focal lengths, as well as extensible for other operating wavelengths, materials, and design techniques. Potential applications include miniaturized imaging systems, AR/VR displays, LiDAR, etc.

## 4 Methods

### 4.1 Gaussian Beam Propagation Model

To theoretically model the lightwave propagation between adjacent metalenses, we utilized the *q* parameter of Gaussian beam. Suppose the *q* parameter of input Gaussian beam is *q*_in_, while the optical distance between adjacent phase profiles is *d*. As property of *q* parameters, the propagation effects through a lens or free space can be both considered as applying four parameters *T*_11_, *T*_12_, *T*_21_, *T*_22_ on *q* as the expression of Equation (4). The values of the four parameters are determined by the focal length *f* or distance *L*, which can be written in the matrix form of **T**_lens_(*f*) or **T**_space_(*L*) in Equation (5). Then, the propagation effects of multiple cascaded layers can be considered as applying the corresponding matrices on *q* in sequence, which are exactly equivalent to applying the multiplication of the matrices on *q*. Thus, the *q* parameter at the output plane (denoted *q*_out_) can be expressed in a cascaded matrix multiplication form as Equation (6), where the output plane is regarded as the top surface of the *N*‐th single‐layer metalens. Finally, the equivalent focal length *f*_e_ of the whole cascaded metalens is defined as the distance between the output surface and the waist of the output beam, which can be calculated with Equation (7). It should be mentioned that the whole cascaded metalens with distance between adjacent single‐layer metalenses is not equivalent to a single lens, and the definition of focal length is similar to the back focal length of an imaging system.

(4) $$q^{\prime }=\frac{T_{11}q_{\mathrm{in}}+T_{12}}{T_{21}q_{\mathrm{in}}+T_{22}}$$

(5) $$T_{\mathrm{lens}}\left(f\right)=\left(\begin{array}{cc} 1 & 0\\ -1/f & 1 \end{array}\right),T_{\mathrm{space}}\left(L\right)=\left(\begin{array}{cc} 1 & L\\ 0 & 1 \end{array}\right)$$

(6) $$T_{\mathrm{whole}}=\prod_{j=1}^{N}T_{\mathrm{space}}\left(L\right)T_{\mathrm{lens}}\left(f_{j,\mu_{j}}\right)$$

(7) fe=−Reqout

### 4.2 Numerical Simulation

The amplitude and phase modulation of rectangular nanopillars with different heights, periods, lengths, and widths are numerically calculated by the Finite Difference Time Domain (FDTD) method. The simulated nanopillar is coated with SU‐8 photoresist. For the eventually used set, the height is 500 nm while the lattice constant is 400 nm, and the length and width are within the range of 80–320 nm. The detailed simulation and design process of single‐layer metalens can be found in Notes S3.1 and S3.2.

The simulation of the focusing property is conducted by light field propagation with Matlab. Details of the simulation can be found in Notes S8.3–S8.5.

### 4.3 Fabrication

First, 500 nm‐thick 𝛼‐Si is deposited on a quartz substrate by PECVD. Then, a layer of Cr as metal hard mask is deposited by electron beam (EB) evaporation, and a layer of SiO_2_ is grown on the Cr layer as an additional hard mask to avoid uncontrollable lift‐off process of Cr. After that, the rectangular patterns are fabricated by electron beam lithography (EBL) and inductively coupled plasma reactive ion etching (ICP‐RIE). The detailed fabrication process without spin‐coating is similar to that of our previous work [50]. Subsequently, the sample is spin‐coated with SU‐8 2002 photoresist at 2000 rpm. Photo‐lithography (without mask) and baking is performed to make a hard spacer layer. A figure describing the fabrication process is presented as Figure S2 of Note S3.3, while the layout and SEM images are presented in Figure S3.

### 4.4 Focusing Property Measurement

The schematic and photograph of the optical setup are presented as Figure S4 of Note S4.1. The input Gaussian beam is filtered to |*D*〉 or |*A*〉 polarization, while the imaging system can move along *z*‐axis as a whole with quantitatively adjusted displacement. Thus, the output *x*‐*y* intensity profiles at different *z* positions are measured and captured by the CCD camera. Among the output intensity profiles, the output plane will be identified by the L mark on the substrate of metalens, while the focal plane will be identified by the optimal focal point. The focal length can be acquired by the *z* displacement between the output plane and the focal plane.

## Author Contributions

Z.M. and X.F. conceived the idea. Z.M. theoretically verified the principle, designed and performed the simulations, experiments, and data analysis. Y.L. contributed significantly to the measurement process. Z.L. and T.T. contributed significantly to the fabrication process. Y.L. contributed to the numerical simulations and measurements. Y.L., K.C., F.L., H.S., and W.Z. provided useful discussions and comments. Z.M. and X.F. wrote the paper. Y.H. revised the manuscript. All authors approved the manuscript.

## Conflicts of Interest

The authors declare no conflicts of interest.

## Supporting information




**Supporting File**: advs75483‐sup‐0001‐SuppMat.pdf.

## Data Availability

All the processed data of this work is provided within the main text and supplementary materials. The raw data is available from the corresponding authors upon request. The codes used for simulation, design, and data processing are available from the corresponding authors upon request.
