# Supplementary material for: Multi‐Channel Electrically Tunable Varifocal Metalens With Compact Multilayer Polarization‐Dependent Metasurfaces and Liquid Crystals
Source: Adv Sci (Weinh). 2026 May 4;13(41):e75483. doi: 10.1002/advs.75483 (PMC13335507; doi:10.1002/advs.75483)
Supplement: Supplementary file 1 — Supporting File: advs75483‐sup‐0001‐SuppMat.pdf. [file ADVS-13-e75483-s001.pdf]

Supplementary Materials for

Multi-channel electrically tunable varifocal metalens with compact multilayer polarization-dependent metasurfaces and liquid crystals

Zhiyao Ma, Yize, Liu, Zhe Li, Tian Tian, Yuxuan Liao, Xue Feng*, Yongzhuo Li, Kaiyu Cui, Fang Liu, Hao Sun, Wei Zhang and Yidong Huang*
*Corresponding author: x-feng@tsinghua.edu.cn (X.F.); yidonghuang@tsinghua.edu.cn (Y.H.)

This file includes:

Supplementary Notes 1: The hyperbolic lens profile 2
Supplementary Notes 2: Calculation and design of equivalent focal lengths 2
2.1 Calculation of focal lengths by Gaussian beam propagation 2
2.2 Design process of the parameters 4
Supplementary Notes 3: Design and fabrication of single-layer metalens 5
3.1 Simulation of rectangle nanopillars 5
3.2 Construction of metalenses 6
3.3 Fabrication of metalenses 7
3.4 Characterization of fabricated metalenses 8
Supplementary Notes 4: Measurement of single metalens 9
4.1. Measurement of focusing property 9
4.2. Imaging demonstration and MTF reconstruction 10
Supplementary Notes 5: Parameters and calibration of customized LC cells 12
Supplementary Notes 6: Stacking and alignment process of cascaded metalens 14
Supplementary Notes 7: Measurement of cascaded metalens 16
Supplementary Notes 8: Discussion of performance 18
8.1 Composition of focusing efficiency and dependence on the number of layers 18
8.2 Non-ideal factors in experiments affecting efficiency and FWHM 19
8.3 Aberration from cascading 20
8.4 Aberration from metasurface design 21
8.5 Aberration from alignment error 23
8.6 List of reported varifocal metalens at optical frequency 24
References 25

Figs. S1 to S13
Tables S1 to S3

Supplementary Notes 1: The hyperbolic lens profile

The hyperbolic lens profile could be written in spatial coordinates (x,y) as[1–5]:

$$\varphi(x,y,f) = -\frac{2\pi}{\lambda}\left(\sqrt{f^2+x^2+y^2}-f\right) \tag{S1}$$

where λ is the wavelength and f is the focal length. It is almost equivalent to the parabolic lens profile in Eq. (1) of the main text in current demonstration, since the N.A. is low and only normal incidence is considered. For more general cases, hyperbolic phase profile eliminates spherical aberration, while parabolic phase profile exhibits wider field-of-view (FOV) for imaging[6].

Supplementary Notes 2: Calculation and design of equivalent focal lengths

2.1 Calculation of focal lengths by Gaussian beam propagation

The equivalent focal length of the whole cascaded structure is calculated by the Gaussian beam propagation model[7]. For a fundamental Gaussian beam propagating along z-axis in vacuum, the complex amplitude of electric field could be written as:

$$\tilde{E}(x,y,z) = \frac{E_0}{w(z)} e^{-ik\frac{r^2}{2}\left[\frac{1}{R(z)}-i\frac{\lambda}{\pi w^2(z)}\right]} e^{-i\left[kz-\arctan\frac{z}{z_R}\right]} \tag{S2}$$

where E_0 is a constant and the other parameters satisfy the following relations:

$$\begin{cases} r^2 = x^2+y^2 \\ k = 2\pi/\lambda \\ w(z) = w_0\sqrt{1+(z/z_R)^2} \\ R(z) = z\left[1+(z_R/z)^2\right] \\ z_R = \pi w_0{}^2/\lambda \end{cases} \tag{S3}$$

where λ is the wavelength; k is the wave vector; w_0 is the beam width at waist; z_R is the Rayleigh range; $w(z)$ is the beam width at z plane; and R(z) is the radius of curvature of the wavefront at z plane. The beam width at a transverse plane is defined by $1/e$ of maximum amplitude. Then, q parameter is defined by the following expression to describe both the curvature of the wavefront and the width of the beam:

$$\frac{1}{q(z)} = \frac{1}{R(z)} - i\frac{\lambda}{\pi w^2(z)} \tag{S4}$$

So that $w(z)$ and R(z) could be readily expressed by $q(z)$:

$$\begin{cases} \dfrac{1}{R(z)} = \mathrm{Re}\left\{\dfrac{1}{q(z)}\right\} \\ \dfrac{1}{w^2(z)} = -\dfrac{\pi}{\lambda}\mathrm{Im}\left\{\dfrac{1}{q(z)}\right\} \end{cases} \tag{S5}$$

For a fundamental Gaussian beam, the relation of q parameters at different z planes could be derived by Eq. (S3) and Eq. (S4). The derived expression is as follows:

$$q(z) = q_0 + z \Rightarrow q(z) + L = q(z + L) \tag{S6}$$

Thus, the change of $w(z)$ and $R(z)$ through propagation could be calculated conveniently by the q parameters. Furthermore, the amplitude profile of the whole Gaussian beam could be determined with given λ and q parameter at any z position. It should be noted that propagation through a medium of thickness t and refractive index n is equivalent to that through a free space of distance $L=t/n$ [8]. Besides, a fundamental Gaussian beam propagating through a thin lens could be described by the following transformation of q parameters:

$$\frac{1}{q_2} = \frac{1}{q_1} - \frac{1}{f} \tag{S7}$$

where q_1, q_2 are the q parameters at the input plane and output plane, and f is the focal length. Eq. (S6) and Eq. (S7) could be rewritten as a unified form to yield Eq. (4)–(6) in the Results section. Then the equivalent focal length f_e of the whole cascaded metalens could be calculated. To be more specific, firstly the input q parameter at metalens 1 is estimated by experiment. The model of the collimator is Thorlabs F260APC-780, whose operation wavelength is λ = 780 nm, the waist diameter of output beam is ~3.33 mm and the focal distance is ~15.11 mm [9]. The distance between the collimator and metalens 1 is ~15 cm. Thus, the following two parameters could be determined:

$$\begin{cases} w_{\text{in}} = 3.33\text{mm}/2 \approx 1.67\text{mm} \\ L_{\text{in,ML1}} = 150\text{mm} - 15.11\text{mm} \approx 135\text{mm} \end{cases} \tag{S8}$$

where w_{in} is the waist radius of the collimated beam, and $L_{\text{in,ML1}}$ is the distance between the waist and metalens 1. Therefore, the following two q parameters could be calculated by the following expression:

$$\begin{cases} q_{\text{in}} = -i\dfrac{\lambda}{\pi w_{\text{in}}^{\ 2}} \\ q_{\text{ML\_in}}^{(1)} = q_{\text{in}} + L_{\text{in,ML1}} \end{cases} \tag{S9}$$

where q_{in} is the q parameter at the waist plane of collimated beam, and $q_{\text{ML\_in}}^{(1)}$ is that at the input plane of metalens 1. Then the value of corresponding $R_{\text{ML\_in}}^{(1)}$ and $w_{\text{ML\_in}}^{(1)}$ could be determined by $q_{\text{ML\_in}}^{(1)}$:

$$\begin{cases} R_{\text{ML\_in}}^{(1)} \approx 935\text{m} \\ w_{\text{ML\_in}}^{(1)} \approx 1.67\text{mm} \end{cases} \tag{S10}$$

It can be seen that the beam width at the input plane of the first metalens is almost equal to that at waist plane, thus the beam is well collimated. Since the diameter of each metalens is 600 μm, the effective beam width at the input plane of the first metalens should be reduced to 300 μm, while the radius of curvature of the wavefront remains constant:

$$\begin{cases} R'^{(1)}_{\mathrm{ML\_in}} \approx 935\mathrm{m} \\ w'^{(1)}_{\mathrm{ML\_in}} \approx 300\mu\mathrm{m} \end{cases} \tag{S11}$$

The corresponding $q'^{(1)}_{\mathrm{ML\_in}}$ is the equivalent q parameter at the input plane of metalens 1. Then, for the cascaded structure, the q parameters at each plane could be calculated by Eq. (S6) and Eq. (S7). The specific expressions are as follows:

$$\begin{cases} 1/q^{(1)}_{\mathrm{ML\_out}} = 1/q'^{(1)}_{\mathrm{ML\_in}} - 1/f^{(1)} \\ q^{(2)}_{\mathrm{ML\_in}} = q^{(1)}_{\mathrm{ML\_out}} + L \\ 1/q^{(2)}_{\mathrm{ML\_out}} = 1/q^{(2)}_{\mathrm{ML\_in}} - 1/f^{(2)} \\ q^{(3)}_{\mathrm{ML\_in}} = q^{(2)}_{\mathrm{ML\_out}} + L \\ 1/q^{(3)}_{\mathrm{ML\_out}} = 1/q^{(3)}_{\mathrm{ML\_in}} - 1/f^{(3)} \end{cases} \tag{S12}$$

where $q^{(j)}_{\mathrm{ML\_in}}$ and $q^{(j)}_{\mathrm{ML\_out}}$ is the q parameter at the input and output plane of metalens j, respectively. L is the equivalent distance between adjacent single-layer metalenses, which should be determined according to the experiment. As mentioned in the main text, the total thickness of one single-layer metalens and one LC cell are d_{ML}~0.54 mm and d_{LC}~1.45 mm, respectively. Since propagation through a medium of thickness t and refractive index n is equivalent to that through a free space of distance $L=d/n$ [8], the equivalent propagation distance between two adjacent single-layer metalenses could be obtained with $n_{\mathrm{SiO_2}}$ ~1.5 as follows:

$$L_{\mathrm{exp}} = \left(d_{\mathrm{LC}} + d_{\mathrm{ML}}\right)/n_{\mathrm{SiO_2}} \sim 1.33 \text{ mm} \tag{S13}$$

It can be verified that Eq. (S12) is equivalent to the matrix multiplication form as Eq. (4)–(6) in the Results section. Finally, the equivalent focal length f_{e} of the whole cascaded metalens could be calculated by Eq. (7) in the Results section, thus obtaining the results of Gaussian beam propagation with L_{exp} = 1.33 mm as well as L = 0 in Fig. 1(d) of the main text.

2.2 Design process of the parameters

At first, according to phase summation method mentioned in the main text and our previous work[10], the reciprocal of cascaded focal length should be the summation of the reciprocals of single focal lengths (see Eq. (3)). Following similar design method in that previous work, a distribution with regular steps regarding the parameter of $1/f$ can be obtained. In short, the dual-channel switchable parameters should be set as (0,1), (0,2), (0,4), …, (0, 2^N-1) for each layer. Therefore, the summation of these parameters can cover all integer values from 0 to 2^N-1, resulting in regular steps. Global shift can be readily introduced by adding it to single layers.

Then, in this work, the $1/f$ parameters are initially chosen as (0,25), (0,50), (100, 200) for each layer (unit: m^{-1}). Thus, the cascaded $1/f$ should be within the range of 100 to 275 with a step of 25 (unit: m^{-1}), corresponding f: 3.6 to 10 mm. At this step, the focal length is an inverse proportional function of the channel number. The minimum step is determined by the layer with minimum difference between $|D\rangle$ and $|A\rangle$ channels.

Then we will consider the thickness and propagation effect between layers. We need to arrange the layers from long to short focal length, so that the propagation effect can be minimized for the former layers. As shown in Fig. 1(d), the focal lengths calculated considering the propagation distance (1.33 mm) are very close to simple phase summation. Finally, the parameters should be slightly adjusted for a relatively smooth trend regarding f. The final $1/f$ parameters are chosen as (0,20), (0,40), (100, 200) for each layer (unit: m^{-1}), corresponding to the f values in Fig. 1(c).

The design principle will be similar for more layers, while more optimization should be conducted for a relatively smooth trend regarding f. In general, the focal length will still be similar to an inverse proportional function of the channel number.

Supplementary Notes 3: Design and fabrication of single-layer metalens

<u>3.1 Simulation of rectangle nanopillars</u>

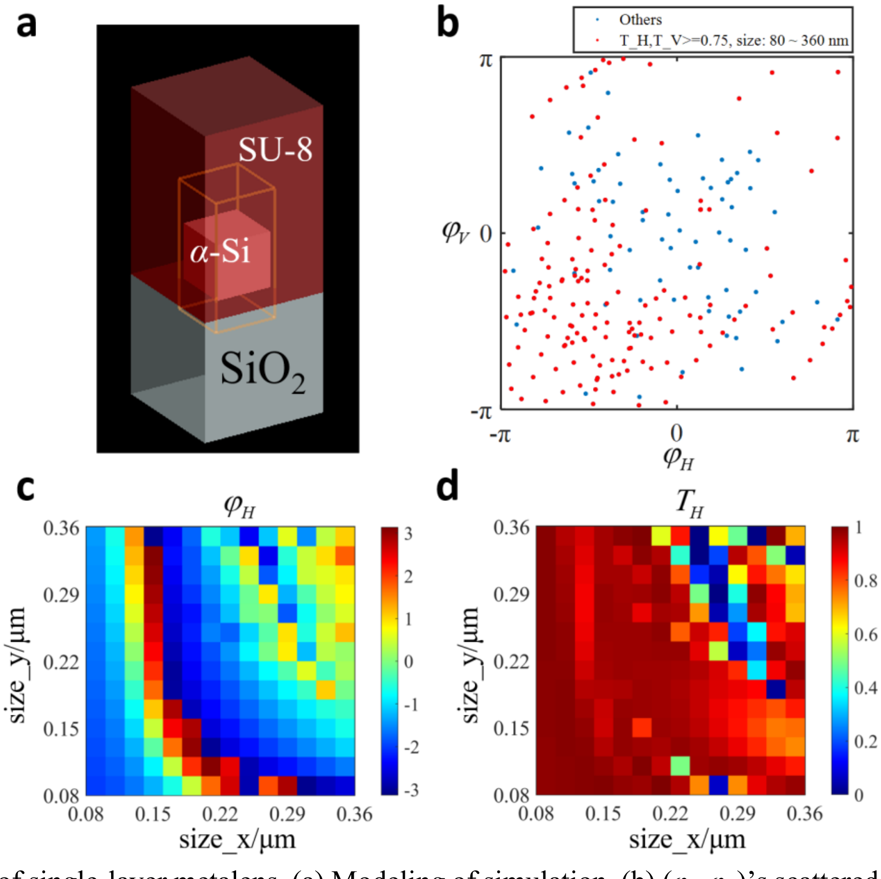

Fig. S1 Design of single-layer metalens. (a) Modeling of simulation. (b) (φ_H,φ_V)'s scattered in the region of $[0,2\pi]\times[0,2\pi]$. The eventually used set of nanopillars is chosen by the condition that T_H,T_V are both higher than 0.75, and marked by red points. The other nanopillars are marked by blue points. (c) The phase modulation values φ_H on input polarization state $|H\rangle$ with respect to the transverse size, while that on $|V\rangle$ could be obtained by mirror-reversal along the line $y=x$ according to the symmetry. (d) The absolute amplitude modulation values T_H on input polarization state $|H\rangle$ with respect to the transverse size, while that on $|V\rangle$ could be obtained by mirror-reversal along the line $y=x$ as well.

The design process of single-layer metalenses is similar to the Supplementary text 3 of our previous work[10], while the operating wavelength in this work is 780 nm. First, the amplitude and phase modulation of rectangular nanopillars with different heights, periods, lengths and widths are numerically calculated by the Finite Difference Time Domain (FDTD) method. The software is Lumerical FDTD supported by Matlab. The modeling is shown in Fig. S1(a), where the rectangular nanopillars are spin-coated with SU-8 photoresist. The boundary conditions are periodic for sides (x-z, y-z plane) and PML for top/bottom (x-y plane). Here, the input polarization state is chosen as $|H\rangle$ and $|V\rangle$ for convenience. The phase modulation values (φ_H,φ_V)'s for all simulated nanopillars are scattered in the region of [0,2π]×[0,2π] and shown in Fig. S1 (b). The eventually used set of nanopillars is chosen by the condition that T_H,T_V are both higher than 0.75 and marked by red points, while the other nanopillars are marked by blue points. As seen in Fig. S1 (b), the red points can fill the region of [0,2π]×[0,2π] with moderate gaps, so that there would be an optimum nanopillar in the set for any required (φ_H,φ_V). The simulated data is presented in Fig. S1 (c)(d). Fig. S1 (c) is the phase modulation values φ_H on input polarization state $|H\rangle$ with respect to the transverse size, while φ_V could be obtained by mirror-reversal along the line y=x according to the symmetry. Fig. S1 (c) shows the absolute amplitude modulation values T_H on input polarization state $|H\rangle$ with respect to the transverse size, while T_H could be obtained similarly by symmetry.

<u>3.2 Construction of metalenses</u>

To construct a full metasurface, firstly, the target dual-phase profiles should be discretized with the resolution of 400 nm, which equals the period of nanopillars. Then, for each pixel, the size (length and width) of the nanopillar is determined by seeking optimal phase modulation values from the set. The error of phase modulation values is defined as:

$$\delta = \sqrt{(\varphi_{H,\text{target}} - \varphi_{H,\text{nanopillar}})^2 + (\varphi_{V,\text{target}} - \varphi_{V,\text{nanopillar}})^2} \quad \text{(S14)}$$

The size with minimum error would be chosen. After all nanopillars are determined, each of them should be rotated 45° along z-axis to transform the input states from $|H\rangle,|V\rangle$ to $|D\rangle,|A\rangle$.

3.3 Fabrication of metalenses

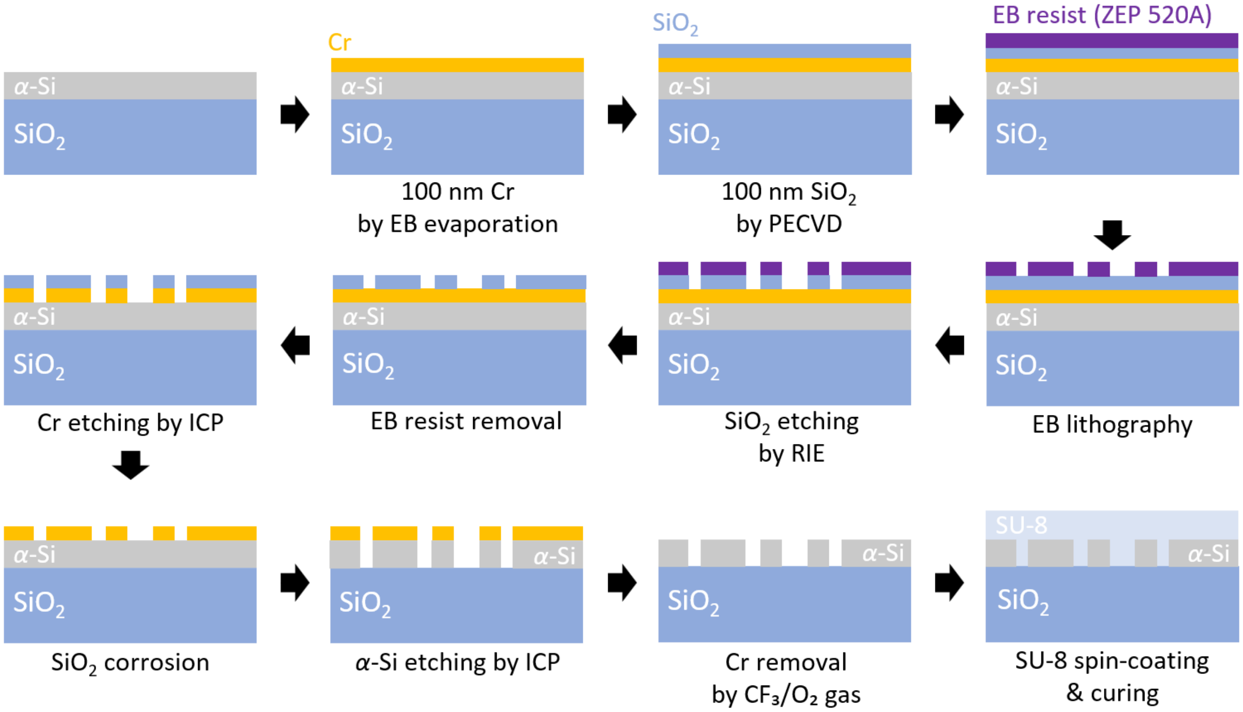

Fig. S2 Fabrication process of single-layer metalens.

3.4 Characterization of fabricated metalenses

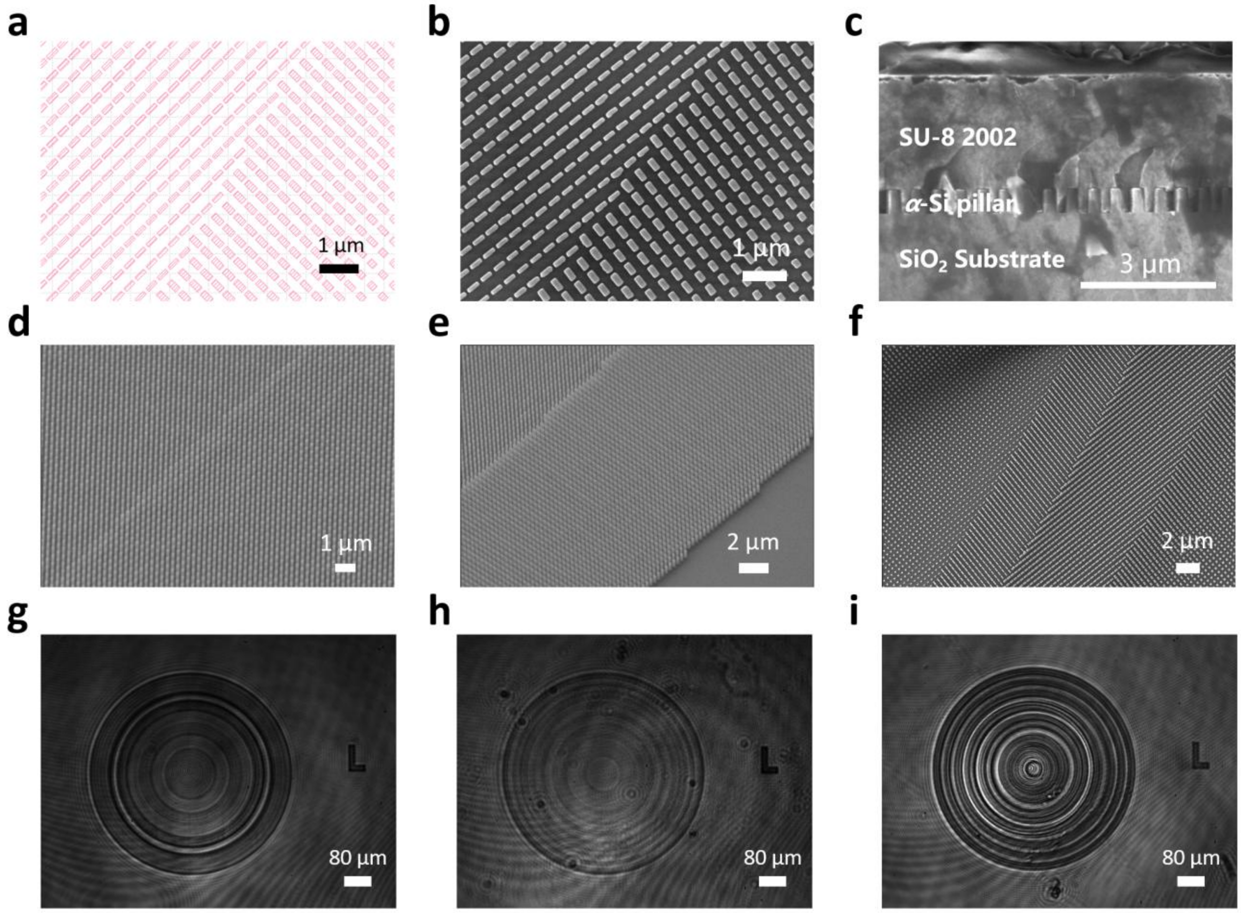

Fig. S3 Characterization of fabricated single-layer metalens. (**a**) Part of the layout of the third metalens. (**b**) SEM image without SU-8 corresponding to the region in (**a**) of the third metalens. (**c**) SEM image of the cross-section with SU-8 spin-coated. (**d**)–(**f**) SEM images without SU-8 of three metalenses, respectively. (**g**)–(**i**) Optical images of three metalenses, respectively.

For the target dual-phase profiles shown in Fig. 1(c) of the main text, a part of the constructed layout of the third metalens is shown in Fig. S3(a). Following the fabrication process above, SEM images without SU-8 are taken. For the region in Fig. S3(a), the corresponding SEM image without SU-8 is presented in Fig. S3 (b). After spin-coating with SU-8, a SEM image of the cross section is shown in Fig. S3 (c). It can be seen that the SU-8 (thickness of ~3 μm) fills all the gaps of the nanopillar (height of ~500 nm). In addition, SEM images with larger views are presented in Fig. S3 (d)–(f) for three metalenses, respectively. For full metalenses, optical images are recorded and presented in Fig. S3 (g)–(i), respectively.

Supplementary Notes 4: Measurement of single metalens

4.1. Measurement of focusing property

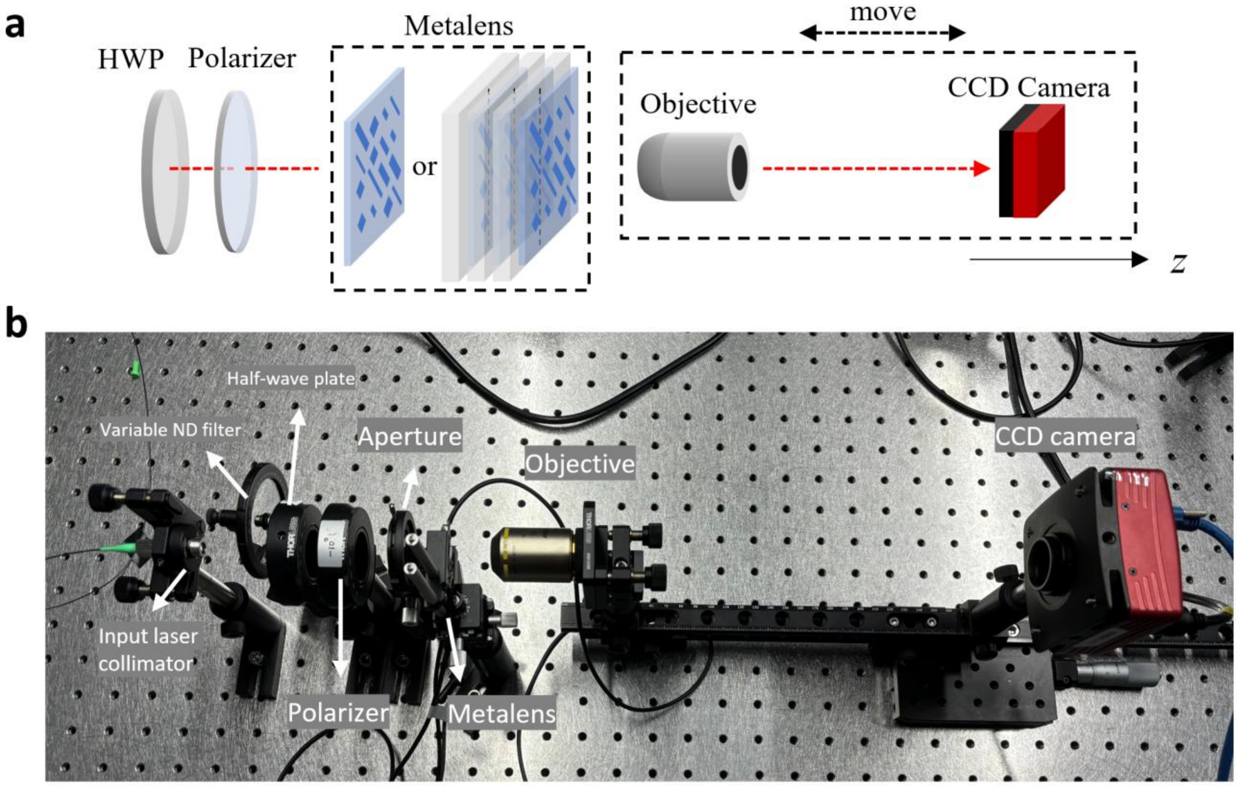

Fig. S4 Optical setup for focusing property measurement. (**a**) Schematic. (**b**) Photograph. This setup is adopted for both single metalens and the cascaded structure.

As mentioned in the main text, the functionalities of all three fabricated single-layer metalenses are measured. The schematic and photograph of the optical setup for measuring the focal length are presented in Fig. S4. This setup is also adopted for measurement of the cascaded structure.

The results of the third metalens are presented in the main text. However, since the focal lengths of the first and second single-layer metalens are relatively long or even $f\to\infty$, the sweeping step of z is chosen as 10 mm, and the focal planes are not quantitatively identified. The replotted intensity profiles at x-z plane of the first single-layer metalens are shown in Fig. S5(a). The output beams with $|D\rangle$ input are not apparently focused, which agrees with the designed focal length of $f\to\infty$. The measured focal length with $|A\rangle$ input is 40~50 mm, whose error from the designed 50 mm is within the 10 mm step of measurement. Also, the replotted intensity profiles at x-z plane of the second single-layer metalens are shown in Fig. S5 (b). The measured focal length with $|A\rangle$ input is also ~∞. While the measured focal length with $|D\rangle$ input is 25~30 mm, whose error from the designed 25 mm is also within the 10 mm step.

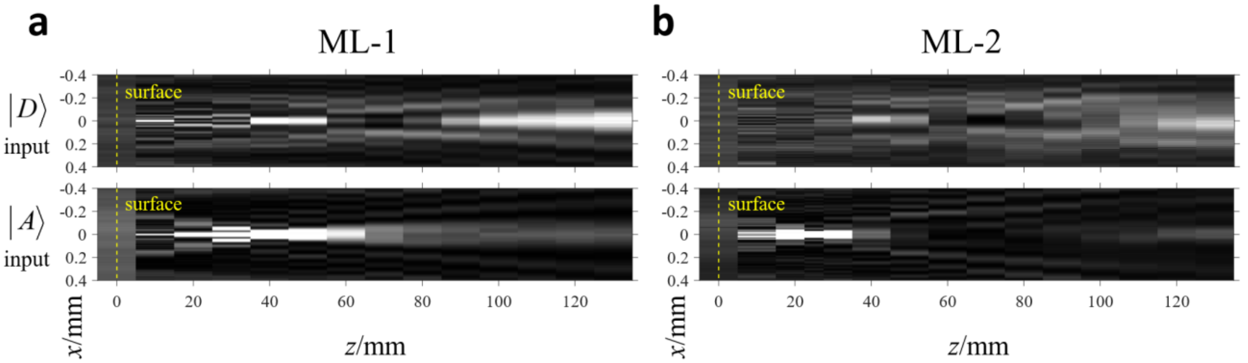

Fig. S5 Measurement of the first and second single-layer metalens. (**a**) Replotted intensity profiles at *x*-*z* plane of the first single-layer metalens. (**b**) Replotted intensity profiles at *x*-*z* plane of the first single-layer metalens.

4.2. Imaging demonstration and MTF reconstruction

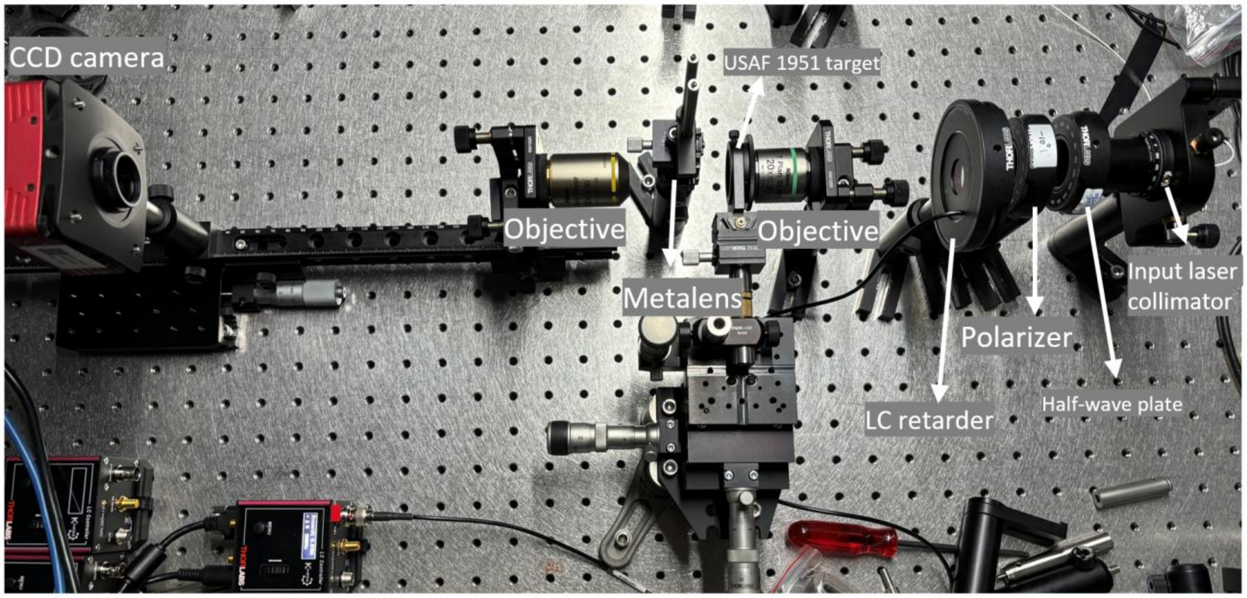

Fig. S6 Photograph of optical setup for imaging measurement. This setup is adopted for both single metalens and the cascaded structure. The LC retarder should be removed while measuring the cascaded structure.

The schematic and photograph of the optical setup for measuring the focal length are presented in Fig. S6. This setup is also adopted for measurement of the cascaded structure. For the MTF and PSF reconstruction of Fig. 6 in the main text, the detailed process is provided as follows.

Firstly, the object is illuminated by a coherent source. Thus, the sampled intensity ESF should be transformed into amplitude ESF. Here, we assume that the field within the sampled region is all in-phase, so that the amplitude ESF is the square root of the intensity ESF. Then, the amplitude Line Spreading Function (LSF) can be obtained by the differential of amplitude ESF.

Next, we would reconstruct the PSF by the LSF. The 2D LSF is the convolution of the 2D PSF with a line delta function $L(x, y) = \delta(x)$:

$$LSF(x,y) = PSF(x,y) * \delta(x) \tag{S15}$$

Hence, the Fourier Transform (FT) of 2D LSF should be:

$$\mathrm{F}\left[LSF(x,y)\right] = c\mathrm{F}\left[PSF(x,y)\right]\delta(y) \tag{S16}$$

where c is a rescaling constant related to FT. Assuming the amplitude PSF has circular symmetry, it can be obtained by the following step: calculate the FT of 1D amplitude LSF, rotate it around the coordinate origin to get a 2D function with circular symmetry, calculate the 2D inverse FT. After that, the commonly used intensity PSF can be obtained by the square of the amplitude PSF.

Then, the intensity LSF can be reconstructed by the reversed process of the last paragraph. MTF can be reconstructed by 1D FT of the intensity LSF[11], while the diffraction-limited ideal MTF is determined by the cutoff frequency 2*NA/λ and a fixed equation[12].

Finally, the reconstructed MTF and ideal MTF can be normalized at the same scale (MTF=1 at spatial frequency f=0). Following similar FT process, reconstructed PSF and the ideal PSF with aligned scale can be obtained, yielding the Strehl ratio by the ratio of their central maximum values.

Supplementary Notes 5: Parameters and calibration of customized LC cells

The LC cells are customized from JCOPITX. Nematic LC molecules are filled in two glass substrates, and the total thickness is measured as 1.45 mm. The following parameters (reference values) of the filled nematic LC molecules are provided by JCOPITX:

Alignment: antiparallel; thickness of LC layer: 12 μm;

Refractive index: extraordinary n_e=1.723, ordinary n_o=1.510, @589nm, 25°C;

Viscosity: 22; Melting Point $T_m \leq$ -40°C, Clearing Point T_{clear} = 103°C;

Rise/fall time: 121.3 ms/3.8 ms, @1064 nm, 25 °C;

For the half-wave retardance at 780 nm required in this work, the rise/fall time can be optimized to be lower than 20 ms by reducing the thickness of the LC layer. Although the cycling stability is not provided, we find that all LC cells are operating with stable retardance values during several months of experiments at room temperature.

We experimentally calibrated all three LC cells used for polarization control. The optical setup for LC calibration is shown in Fig. S7. For each LC cell, the voltages corresponding to 0 and π phase retardance are calibrated. Firstly, the input polarization state at the LC is filtered to $|D\rangle$. Since the slow axis of LC is fixed in the direction of θ = 0, the polarization state would remain $|D\rangle$ or be varied to $|A\rangle$ according to 0 or π phase retardance, respectively. Thus, another polarizer is put after the LC to analyze the polarization states. The output is observed while sweeping the voltage on LC. If the output is eliminated by a polarizer with θ = 135° (corresponding to $|A\rangle$), the output polarization state should be $|D\rangle$ and the phase retardance of LC should be 0. If the output is eliminated by a polarizer with θ = 45° (corresponding to $|D\rangle$), the output polarization state should be $|A\rangle$ and the phase retardance of LC should be π.

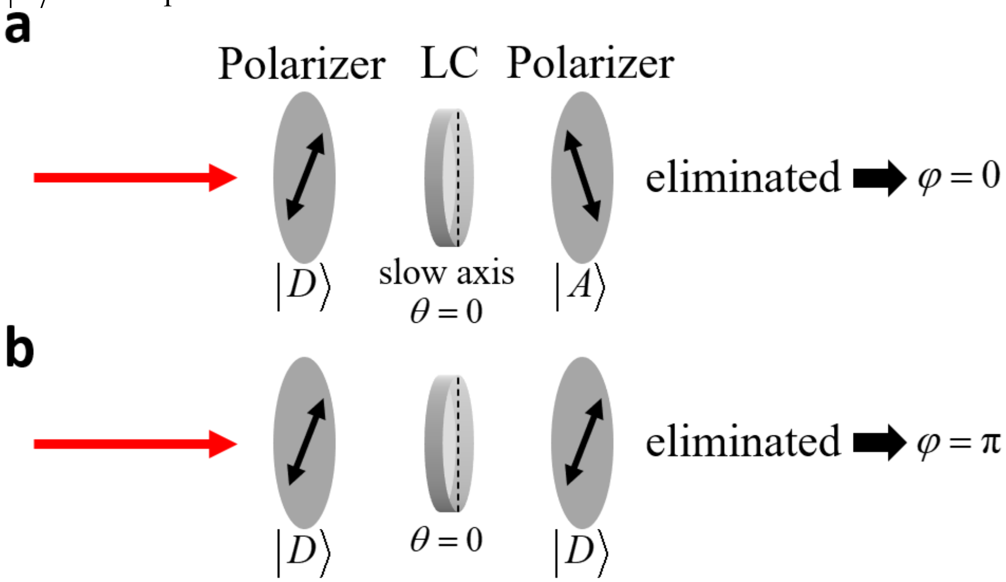

Fig. S7 Calibration setup of LC. (**a**) Calibration of the voltage corresponding to 0 phase retardance. (**b**) Calibration of the voltage corresponding to π phase retardance.

It should be noted that the phase retardance of LC varies spatially since the thickness of LC layer is not uniform, which is also mentioned in the section of Methods. Therefore, the calibration of LC should be conducted while being attached to the cascaded structure, so that the corresponding region around the metalens could be calibrated. Furthermore, the calibration of the second/third LC should be conducted while the other LC(s) are under the voltage of 0 phase retardance.

The LCs are driven by 2 kHz square wave voltage with Thorlabs KLC101. The calibrated V_{rms} (root mean square voltages) values are listed in Table S1. In addition, for each of the eight focal lengths in the cascaded structure, the required phase retardances of each LC cell can be immediately obtained by the input state $|D\rangle$ and required polarization of each layer. The specific configurations are provided in Table S2.

Table S1 Voltage results of LC calibration

Phase retardance	LC_1, V_{rms}	LC_2, V_{rms}	LC_3, V_{rms}
π	1.29 V	1.20 V	1.28 V
0 (2π)	1.55 V	1.49 V	1.55 V

Table S2 Corresponding phase configuration to the polarization combination of eight channels

Polarization	*DDD*	*ADD*	*DAD*	*AAD*	*DDA*	*ADA*	*DAA*	*AAA*
LC retardance	0,0,0	π,π,0	0,π,π	π,0,π	0,0,π	π,π,π	0,π,0	π,0,0

Supplementary Notes 6: Stacking and alignment process of cascaded metalens

The photographs of LC cell and single-layer metalens are shown in Fig. S8(a)(b), respectively. The implementation process of the three-layer cascaded metalens is shown in Fig. S9. To start with, since the transverse size of LC cells (2 cm × 2 cm) is larger than that of metalens substrates (1 cm × 1 cm), the first metalens is attached to the right area of the first LC cell. While the second LC cell is attached to the first metalens with a large displacement to the right relative to the first LC cell, so that the tape attaching and wire connecting would be convenient. Next, the second metalens is attached to the second LC cell and roughly overlaps with the first metalens. After that, the third LC cell is attached to the second metalens and overlaps with the first LC cell. Finally, the third metalens is attached and roughly overlaps with the first two metalenses. In the process, tapes are used to roughly fix the devices. Then a holder (Thorlabs CH2B) is used to firmly fix the three LC cells on the sides (see Fig. 3).

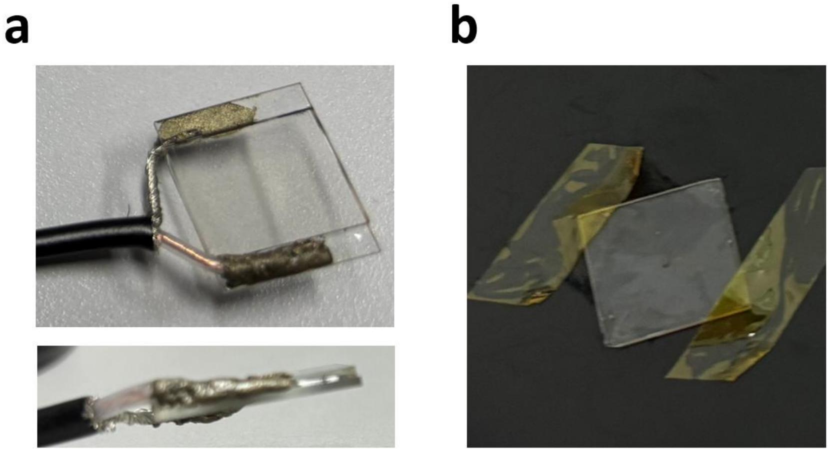

Fig. S8 Photographs of LC cell and single-layer metalens. (**a**) LC cell customized from JCOPTIX, total size 2 cm × 2 cm × 1.45 mm. (**b**) Single-layer metalens with SU-8 spin-coated, effective diameter 600 μm, total size 1 cm × 1 cm × 0.54 mm.

For more precise alignment, the area of metalenses and the L markers should be imaged to qualitatively observe the relative displacement. Since the metalens substrates are smaller than LC cells, they are fixed by tapes that are not as firm as the holder. Thus, the attached location of the metalenses could be slightly adjusted by tweezers until they align well. The image of the aligned of metalenses is presented as follows.

The alignment of three single-layer metalenses is characterized with similar optical setup in Fig. S4. By imaging at different z positions, the surface of three single-layer metalenses could be determined by the L marker as z = 0.75 mm, 2.07 mm, 3.46 mm, respectively. As shown in Fig. S10(a), the error of transverse positions of three L markers at the image planes is within 0.5 mm. Since the designed length and width of the L marker are 80 μm and 50 μm, it could be calculated that the error at the object planes is within 34 μm. Fig. S10 (b) shows a full image at the surface of the third metalens, where it can be qualitatively observed that the three metalenses are aligned in the same transverse position.

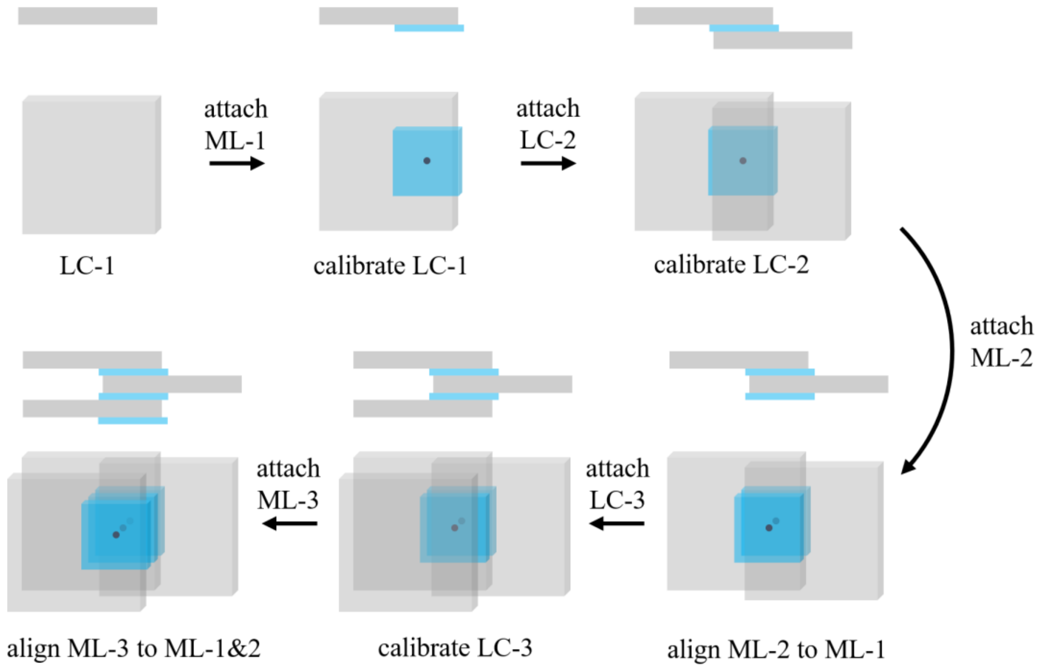

Fig. S9 Schematic of the implementation process of the three-layer cascaded metalens. The liquid crystal (LC) cells and single-layer metalens (ML) are alternately attached. The calibration of LCs and alignment of MLs are conducted at corresponding steps.

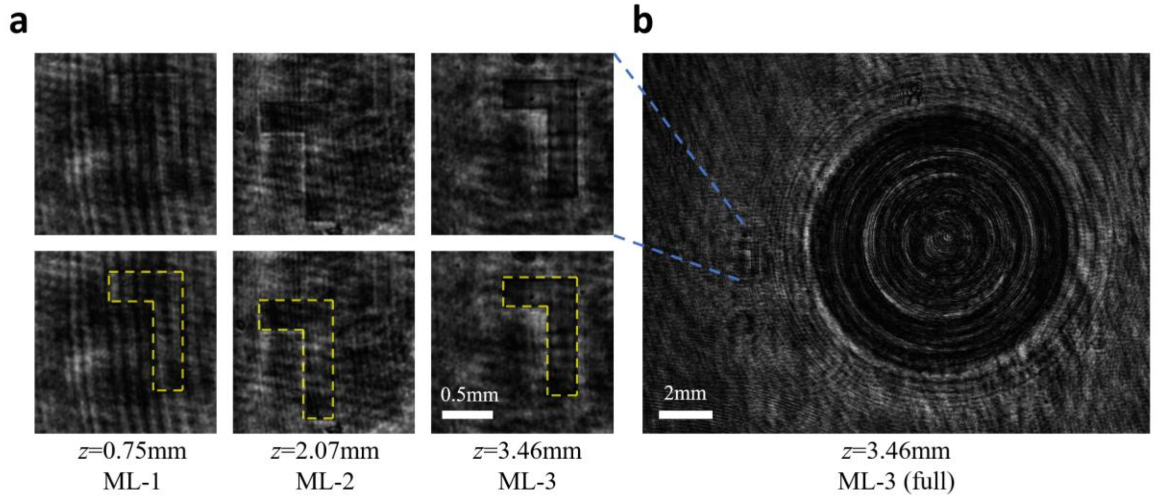

Fig. S10 Alignment of three single-layer metalenses. The scale bars correspond to the size at image plane. (**a**) Experimental imaging of the L marker at the surface of each single-layer metalens, where the corresponding *z* positions are noted. First row: original images. Second row: the L markers are sketched by yellow dashed lines. (**b**) A full image at the surface of the third metalens.

Supplementary Notes 7: Measurement of cascaded metalens

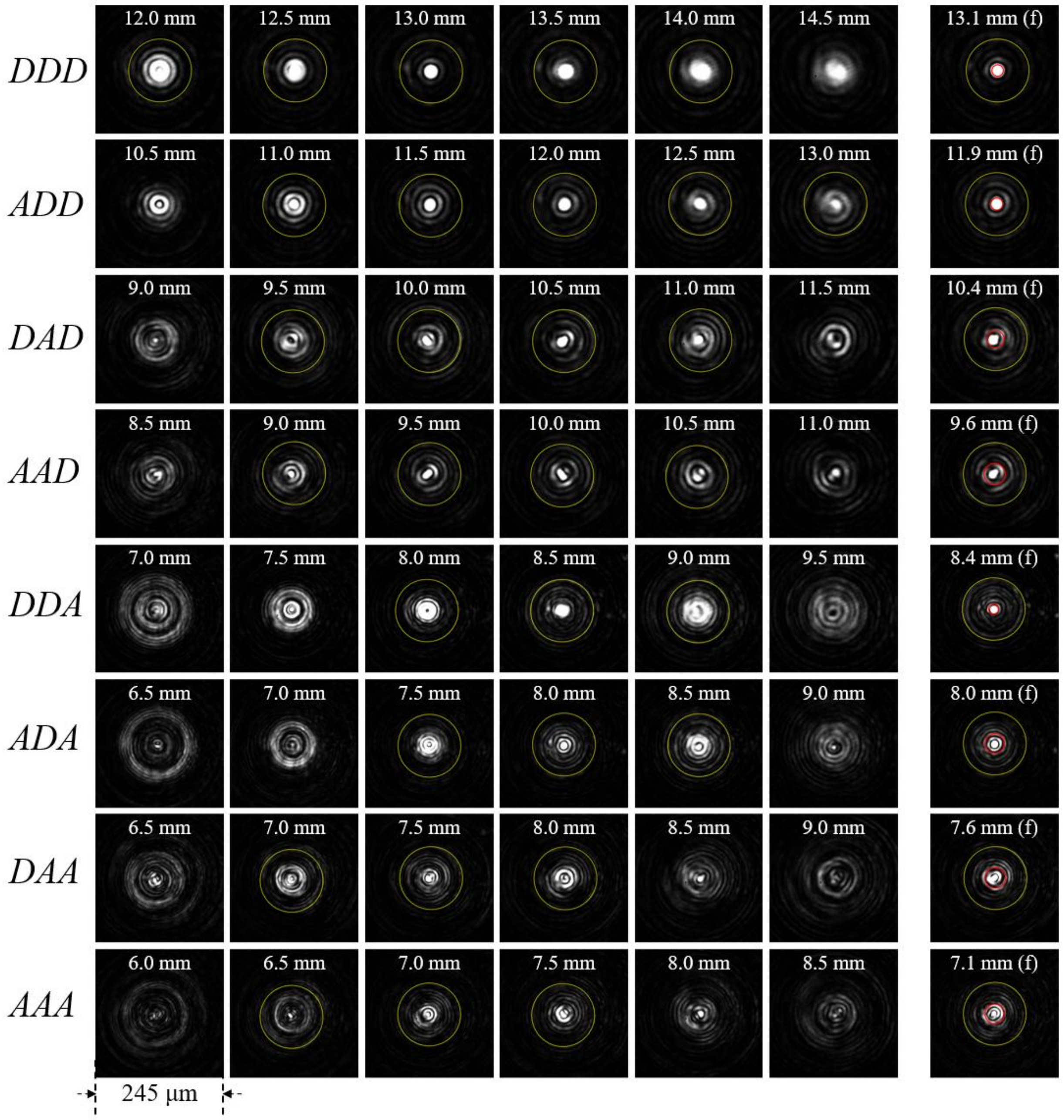

Fig. S11 Measured *x*-*y* intensity profiles of eight-channel cascaded metalens. The displacement along *z*-axis is noted on each profile. The region of FWHM is marked by red circles, while the area of integration for efficiency calculation is marked by yellow circles. The scale corresponds to the size at object plane.

Fig. S11 shows part of the measured intensity profiles at *x*-*y* plane of eight-channel cascaded metalens. For each channel, only the intensity profile at focal plane and six nearest positions are shown. The intensity profiles at *x*-*z* plane shown in Fig. 4(a) of the main text are replotted from all intensity profiles at *x*-*y* plane within the range of z=0 to z=14 mm. Besides, the FWHM is

measured by 2-dimensional Gaussian fitting of the intensity profile at the focal plane and marked by red circles in the rightmost column of Fig. S11. The efficiency is defined as the ratio of the total intensity around the focal spot to the intensity of the input. Since the focal intensity (numerator) is highly sensitive to the area of integration, the diameter of integration is chosen as a fixed value of 120 μm, which is according to 3 times the maximum FWHM (~40 μm) among all channels. The area of integration is marked by yellow circles in Fig. S11. It should be noted that some profiles at focal plane are overexposed. Thus, the profiles among z positions within ±10% deviation from focal length are all considered for calculation. The profiles to be calculated are all marked by the yellow circles in Fig. S11. Then, for each channel, the final focal intensity is chosen as the maximum value among the calculated profiles.

Supplementary Notes 8: Discussion of performance

8.1 Composition of focusing efficiency and dependence on the number of layers

First, the composition of focusing efficiency is analyzed and its dependence on the number of layers is estimated. Here, we define the transmittance t as the intensity ratio between the output and input plane, while the transmitted focusing efficiency η_f is the intensity ratio between the focal plane and the output plane. Thus, the total efficiency η is the product of t and η_f. The definitions are expressed as follows:

$$t = I_{\text{out\_plane}} / I_{\text{in}}$$
$$\eta_{\text{f}} = I_{\text{focal}} / I_{\text{out\_plane}} \tag{S17}$$
$$\eta = t\eta_{\text{f}}$$

For a single metalens, the transmittance t_{single} should be the product of the transmittance through the substrate and that through the nanostructure:

$$\eta_{\text{single}} = t_{\text{single}}\eta_{\text{f,single}} = t_{\text{sub}}t_{\text{ML}}\eta_{\text{f,single}} \tag{S18}$$

In our scheme, the designed focal lengths of the former layers are much longer than the final tunable range, while the focal lengths of the last layer are within the final range. That is to say, the main phase gradient is contributed by the last layer, as well as the focusing loss regarding η_f. For the former layers, the output will be measured far away from their focal spot, so that the loss through transmittance without focusing should be mainly considered. As a result, the total focusing efficiency η_{total} can be estimated by the product of transmittance of each metalens, t_{LC} (the transmittance of LCs), and η_f of the last metalens:

$$t_{\text{total}} = \prod_{k=1}^{N} t_{\text{LC},k}t_{\text{sub},k}t_{\text{ML},k}$$
$$\eta_{\text{f,total}} = \eta_{\text{f},N} \tag{S19}$$
$$\eta_{\text{total}} = t_{\text{total}}\eta_{\text{f,total}} = \left(\prod_{k=1}^{N} t_{\text{LC},k}t_{\text{sub},k}t_{\text{ML},k}\right)\eta_{\text{f},N}$$

This estimation can be supported by the measured results in this work. For the whole cascaded structure, t_{total} is within the range of 16% to 26% and $\eta_{\text{f,total}}$ is within the range of 32% to 46%, yielding the total efficiency within the range of 5% to 10%. We have measured the efficiency of the third metalens with the same process (see Supplementary Notes 7) for reference values of single metasurface. For $|D\rangle$ channel, t_3 is 61.7% and $\eta_{\text{f},3}$ is 50.4%; for $|A\rangle$ channel, t_3 is 55.8% and $\eta_{\text{f},3}$ is 41.7%. Given t_{LC}~85%, N=3, and all layers have the same transmittance, the value of t_{total} can be estimated by Eq. (S19) as:

$$t_{\text{total}} \sim 0.617^3 \times 0.85^3 = 14.4\% \tag{S20}$$

which is very close to the measured results (16% to 26%). Besides, Eq. (S19) can be supported since measured t_{total} is significantly lower than t_3 due to cascading, while measured $\eta_{\text{f,total}}$ is close

to $\eta_{f,3}$. To conclude, the total efficiency is mostly a product of transmittance through each layer, which proves that our cascading scheme would not introduce additional loss.

8.2 Non-ideal factors in experiments affecting efficiency and FWHM

With each component in Eq. (S19), the non-ideal factors in experiments affecting the efficiency can be investigated accordingly, along with possible solutions and expected improvement:

(**i**) t_{LC}: The measured transmittance through an LC cell is ~85%, which mainly comes from reflection at LC substrates and scratches/pollution on the substrates during stacking. Besides, polarization crosstalk through a single LC cell is ~150:1 for $|D\rangle$ to $|D\rangle$ polarization control, and ~90:1 for $|D\rangle$ to $|A\rangle$ polarization control.

Solution: With proper AR coating, the transmittance through LC can be increased to higher than 98% [13]. The stacking process can be advanced by automated motorized stages for alignment and UV glue for attachment. To reduce the polarization crosstalk, the alignment and packaging process of custom LC cells can be optimized for uniform thickness and refractive index.

(**ii**) t_{sub}: The measured transmittance through a metalens substrate is ~90%, which also comes from reflection and scratches/pollution during stacking.

Solution: AR coating and advanced stacking process for higher than 98%.

(**iii**) t_{ML}: Since the measured total transmittance through a metalens is 50~60%, it can be calculated that transmittance through the nanostructure is 56~57%. This comes from the metalens design and fabrication, *e.g.* the reflection/scattering at single nanopillars, crosstalk between adjacent nanopillars, *etc*. In addition, the spacer layer made by spin-coating is non-uniform.

Solution: Inverse design, topological optimization, and iteration of fabrication process. Optimizing the model of photoresist and parameters of spin-coating. The efficiency of a single nanopillar is potentially to reach 95% with P-B phase design[14].

(**iv**) η_f: The measured transmitted focusing efficiency through the third metalens is 40~50%, which means more than half of transmitted light is not focused to the focal spot. The reasons include scattering, non-ideal phase profile, polarization crosstalk through metalens, *etc*.

Solution: Inverse design, topological optimization, and iteration of fabrication process. The focusing efficiency of single layer can reach 60%~80% in a reported work[15].

Supposing the efficiency could be optimized as above, and according to Eq.(S21), the expected efficiency can be increased to $(0.98\times0.98\times0.8)^N\times0.8$. Here, both the transmittance of single metalens and the transmitted focusing efficiency are expected to be 80% (total efficiency 64%) as a more conservative estimation. For example, if N=6, the expected efficiency will be 20.1%, and 2^6=64 focal length values are sufficient for most applications.

Most factors mentioned above would also affect the FWHM, including multiple reflection, scattering, polarization crosstalk, non-ideal phase profile, etc. In addition, FWHM is affected by the error from measurement and fitting.

To further investigate the aberration from cascading, metalens design and alignment, simulations by light field propagation with Matlab are conducted in the following Section 8.3 to 8.5. Finally, a list of reported varifocal metalens at optical frequency is provided in Section 8.6.

8.3 Aberration from cascading

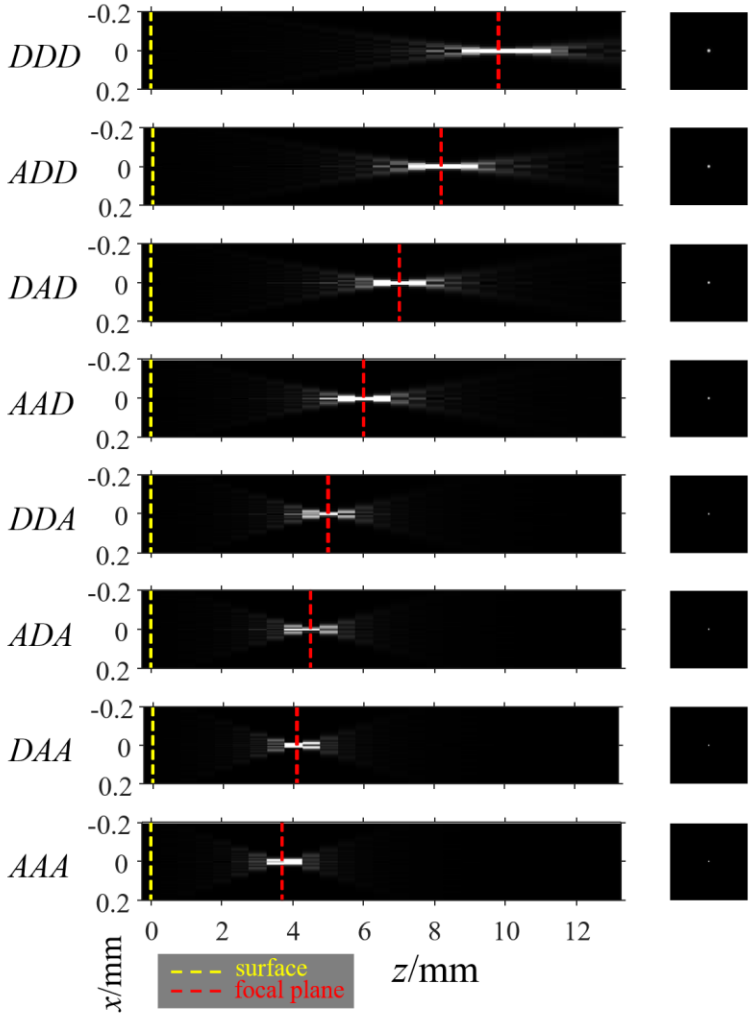

Fig. S12 Simulation of focused *x*-*z*-plane intensity of the cascaded metalens. The *x*-*y*-plane intensity at focal plane is provided at the right column.

The focused *x*-*z* intensity of the cascaded metalens is simulated by Fourier Transform based light propagation in Matlab. Here, the sampling resolution is 2048×2048 with 400 nm pixel size (identical to the meta-atom size) at *x*-*y* plane. The incident is a Gaussian beam with 1500 µm radius. The distance between adjacent layers is 1.33 mm. All three metasurfaces are set as ideal profiles

according to the expected polarization states of each channel. For each channel, after the third layer, the propagation distance *z* is sampled from 0 mm to 13 mm with 0.5 mm step. The obtained *x-z* plane intensity is shown in the left column of Fig. S12 Simulation of focused *x-z-plane* intensity of the cascaded metalens. The *x-y-plane* intensity at focal plane is provided at the right column.. Then, a coarse focal length is obtained at the plane with maximum intensity. Next, a fine sampling is conducted within ±0.7 mm around the coarse focal length with 0.1 mm step. After that, the fine focal length and the *x-y* intensity at focal plane (right column of Fig. S12 Simulation of focused *x-z-plane* intensity of the cascaded metalens. The *x-y-plane* intensity at focal plane is provided at the right column.) can be obtained, as well as the FWHM. The focal length and FWHM are plotted in Fig. 4 (b) and (c) of the main text, respectively.

8.4 Aberration from metasurface design

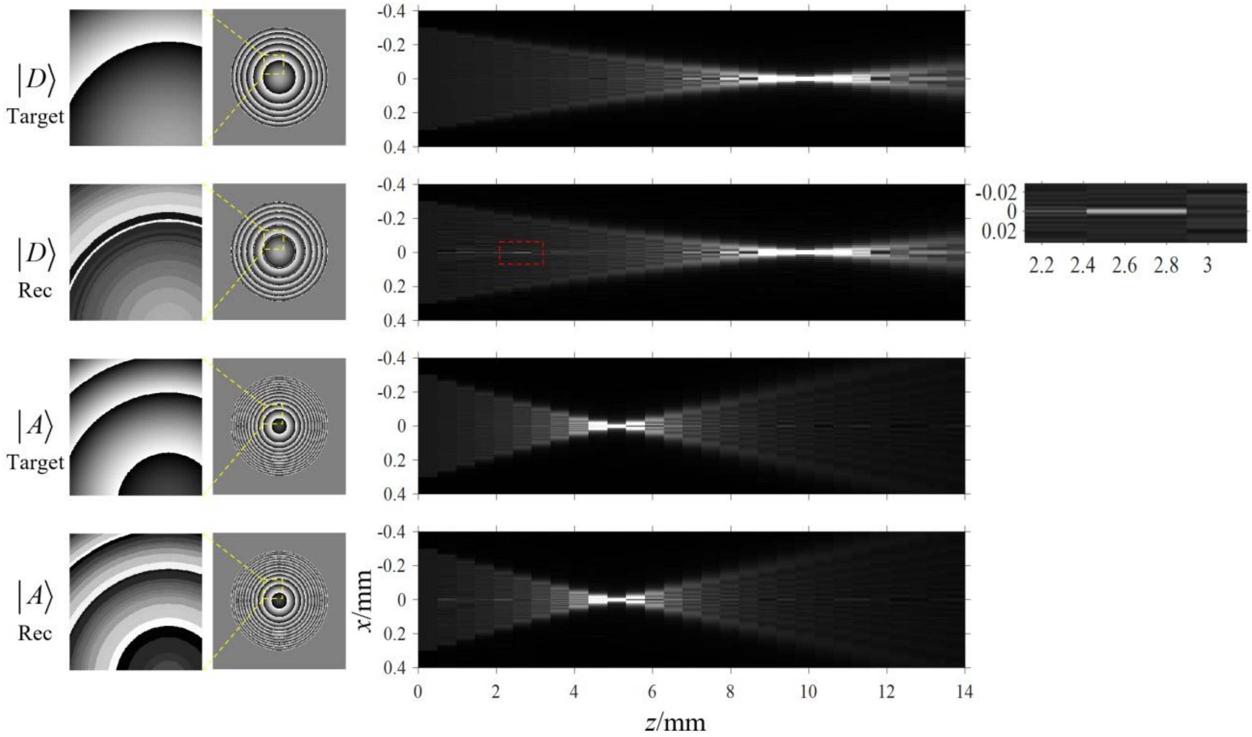

Fig. S13 Simulation of focused *x-z*-plane intensity of the third metalens. The input beam propagates through target and reconstructed (Rec) phase profiles of both polarization states. Zoomed pictures and full pictures of phase profiles are shown in the left part. For the reconstructed profile of $|D\rangle$ input, a zoomed picture of a stray-light focal spot is shown in the right part.

The focused *x-z* intensity of the third metalens is simulated. The phase profiles are obtained in the design process of Supplementary Notes 2.2. The input wave propagates through the target and reconstructed (Rec) phase profiles with both input polarization states. Zoomed pictures and full pictures of phase profiles are shown in the left part of Fig. S13. It can be seen that the reconstructed profiles are discrete approximations of target ones. For the simulated focused *x-z-*

plane intensity of reconstructed profile with $|D\rangle$ input, a zoomed picture of an unexpected stray-light focal spot at ~2.6 mm from the surface is shown in the right part, which is similar to the measured results in Fig. 2(b) of the main text. While the stray-light focal spot is not seen in the simulated results of target phase profile. Therefore, such a stray-light focal spot is an aberration effect from the non-ideal reconstructed phase profile. It can be addressed by employing a larger set of nanopillars in the design process to reduce the reconstruction error of phase profile.

8.5 Aberration from alignment error

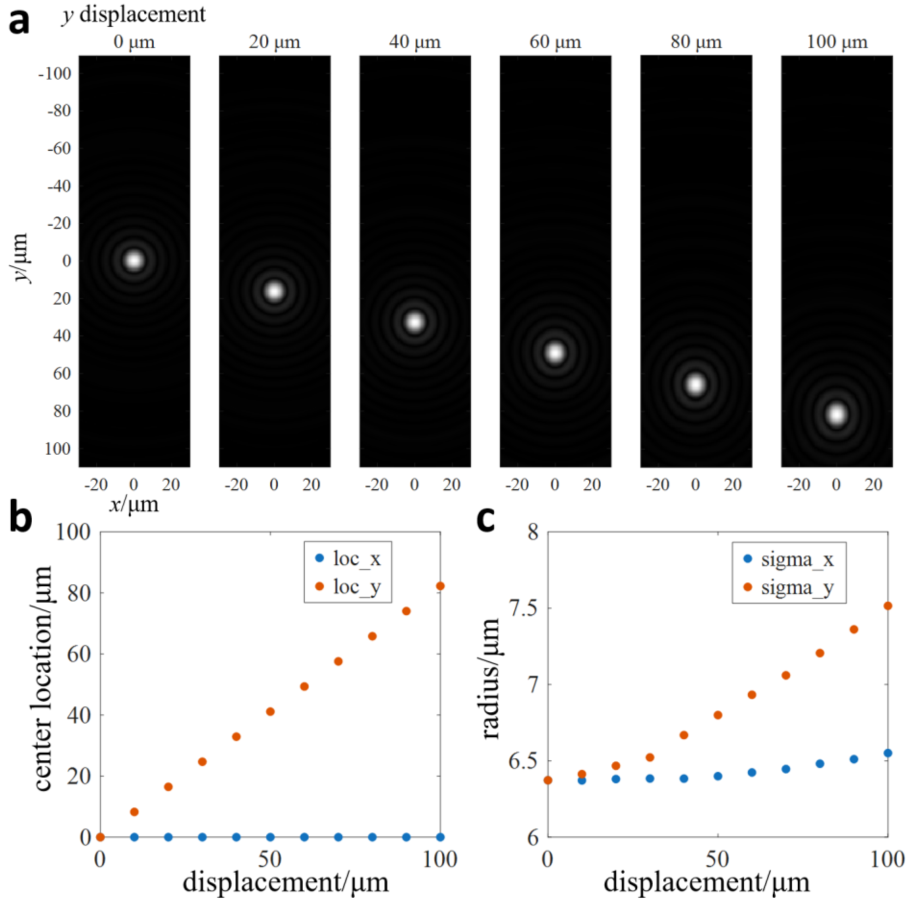

Fig. S14 Simulation of focused *x*-*y*-plane intensity of two cascaded metalens with alignment error 0 to 100 μm. (**a**) Simulated *x*-*y*-plane intensity at the focal spot *z*=4.1 mm; (**b**) the center and (**c**) radius of the beam obtained by 2D Gaussian fitting.

The effect of alignment error is investigated by simulation of two cascaded metalenses. The first metalens is omitted, and the reconstructed phase profiles of the second and third metalenses with both $|A\rangle$ inputs are cascaded. The focal lengths are 25 mm and 5 mm, respectively, while the distance is 1.5 mm. Then, the alignment error is introduced by the displacement of the third metalens along *y*-axis, within the range of 0 to 100 μm. The simulated *x*-*y*-plane intensity at the focal spot *z*=4.1 mm is shown in Fig. S14(a). Then, the center and radius of the beam are obtained by 2D Gaussian fitting and shown in Fig. S14(b)(c). It can be seen that the focal spot would move along *y*-axis linearly to the displacement value, while the size of the focal spot would only moderately spread along *y*-axis. At the displacement of 34 μm (experimental value), the spot would move ~30 μm, and the *y* size would spread ~5% larger than the case of no displacement. Therefore, the alignment error in our demonstration would not significantly affect the performance.

8.6 List of reported varifocal metalens at optical frequency

Table S3 List of reported varifocal metalens at optical frequency.

Tuning method	Tuning mechanism & Reference	Operating wavelength	Range of focal lengths	Number of focal lengths	Diameter	efficiency	FWHM	speed	Imaging demo	FOV
Mechanical	Stretching substrate[16]	1550 nm	50 to 65 mm	continuous	6 mm	65.2%	34.4±1.1 µm	33±3 ms	NO	N/A
	Stretching substrate[17]	633 nm	150 to 250 µm	continuous	75 µm	N/A	~1 to 2 µm	N/A	NO	N/A
	MEMS[18]	910 nm	627 to 824 µm	continuous	300 µm	40 to 50%	~ 40 µm	~kHz	YES	40°
	Rotational Moiré profiles[19]	532 nm	10 to 125 mm	continuous	1.6 mm	~35%	7.5 µm	N/A	YES	N/A
	Rotational Moiré profiles[20]	532 nm	3.7 to 33.2 mm	continuous	1 mm	14.2%	N/A	N/A	YES	N/A
Thermal	GST phase-change material[21]	3.1 µm	0.5 to 1 mm	2	~600 µm (cylindrical)	10%	N/A	N/A	NO	N/A
	Sb_2Se_3 phase-change material[22]	1550 nm	50 to 120 µm	2	35 µm	5.74%	2.52 µm	N/A	NO	N/A
Electrical	LC[23]	808 nm	17 to 20 mm	continuous	590 µm	8.2 to 11.8%	24 to 30 µm	N/A	YES	N/A
	LC polarization[14]	633 nm	3.7 to 7.5 mm	2	1.5 mm	44%	~3 to 5 µm	~ ms	YES	N/A
	LC polarization[15]	450, 532, 635 nm	50 to 100 µm	2 (each wavelength)	100 µm	60 to 80%	~1 to 2 µm	N/A	YES	N/A
	**LC polarization (This work)**	780 nm	3.6 to 9.6 mm	8	600 µm	~5 to 10%	~20 to 40 µm	~ 100 ms	YES	>4°

References

1. Chen, X. *et al.* Dual-polarity plasmonic metalens for visible light. *Nat. Commun.* **3**, 1198 (2012).
2. Wang, S. *et al.* Broadband achromatic optical metasurface devices. *Nat. Commun.* **8**, 187 (2017).
3. Chen, W. T. *et al.* A broadband achromatic metalens for focusing and imaging in the visible. *Nat. Nanotechnol.* **13**, 220–226 (2018).
4. Wang, S. *et al.* A broadband achromatic metalens in the visible. *Nat. Nanotechnol.* **13**, 227–232 (2018).
5. Khorasaninejad, M. *et al.* Metalenses at visible wavelengths: Diffraction-limited focusing and subwavelength resolution imaging. *Science* **352**, 1190–1194 (2016).
6. Yang, F. *et al.* Wide field-of-view metalens: a tutorial. *Adv. Photonics* **5**, (2023).
7. Svelto, O. Chapter 4.7 Gaussian Beams. in *Principles of Lasers* P150-159 (Springer US, Boston, MA, 2010). doi:10.1007/978-1-4419-1302-9.
8. Ooya, T., Tateiba, M. & Fukumitsu, O. Transmission and reflection of a gaussian beam at normal incidence on a dielectric slab. *J. Opt. Soc. Am.* **65**, 537 (1975).
9. Fixed Focus Collimation Packages: FC/APC Connectors. https://www.thorlabs.com/newgrouppage9.cfm?objectgroup_ID=1696.
10. Ma, Z. *et al.* Electrically switchable 2N-channel wave-front control for certain functionalities with N cascaded polarization-dependent metasurfaces. *Nat. Commun.* **15**, 8370 (2024).
11. Optical transfer function - Wikipedia. https://en.wikipedia.org/wiki/Optical_transfer_function.
12. Introduction to Modulation Transfer Function. https://www.edmundoptics.com/knowledge-center/application-notes/optics/introduction-to-modulation-transfer-function/.
13. Half-Wave Liquid Crystal Variable Retarders / Wave Plates. https://www.thorlabs.com/newgrouppage9.cfm?objectgroup_id=6179.
14. Badloe, T., Kim, I., Kim, Y., Kim, J. & Rho, J. Electrically Tunable Bifocal Metalens with Diffraction-Limited Focusing and Imaging at Visible Wavelengths. *Adv. Sci.* **8**, 2102646 (2021).
15. Ou, X. *et al.* Tunable Polarization-Multiplexed Achromatic Dielectric Metalens. *Nano Lett.* **22**, 10049–10056 (2022).
16. She, A., Zhang, S., Shian, S., Clarke, D. R. & Capasso, F. Adaptive metalenses with simultaneous electrical control of focal length, astigmatism, and shift. *Sci. Adv.* **4**, eaap9957 (2018).
17. Ee, H.-S. & Agarwal, R. Tunable Metasurface and Flat Optical Zoom Lens on a Stretchable Substrate. *Nano Lett.* **16**, 2818–2823 (2016).
18. Arbabi, E. *et al.* MEMS-tunable dielectric metasurface lens. *Nat. Commun.* **9**, 812 (2018).
19. Luo, Y. *et al.* Varifocal Metalens for Optical Sectioning Fluorescence Microscopy. *Nano Lett.* **21**, 5133–5142 (2021).
20. Song, Y. *et al.* Three-dimensional varifocal meta-device for augmented reality display. *PhotoniX* **6**, 6 (2025).
21. Yin, X. *et al.* Beam switching and bifocal zoom lensing using active plasmonic metasurfaces. *Light Sci. Appl.* **6**, e17016–e17016 (2017).
22. Wang, M. *et al.* Varifocal Metalens Using Tunable and Ultralow-loss Dielectrics. *Adv. Sci.* **10**, 2204899 (2023).
23. Bosch, M. *et al.* Voltage-Tunable Multifunctional Zoom Imaging Metalenses. *ACS Photonics* **12**, 728–736 (2025).
